# Factors Impacting the Nuclear Magnetic Resonance Spectra of Electrolyte Adsorbed in Layered Metal–Organic Frameworks

**DOI:** 10.1021/acs.jpcc.5c04963

**Published:** 2025-11-19

**Authors:** Chloe J. Balhatchet, Jamie W. Gittins, Ieuan D. Seymour, Ryan J. Bragg, Seung-Jae Shin, Teedhat Trisukhon, Thomas Kress, John M. Griffin, Alexander C. Forse

**Affiliations:** † Yusuf Hamied Department of Chemistry, 2152 University of Cambridge, Lensfield Road, Cambridge CB2 1EW, U.K.; ‡ Advanced Centre for Energy and Sustainability (ACES), Department of Chemistry, 1019 University of Aberdeen, Meston Walk, Aberdeen AB24 3UE, U.K.; § Department of Chemistry, University of Lancaster, Lancaster LA1 4YW, U.K.; ∥ Department of Chemistry, 2707 University of Warwick, Coventry CV4 7AL, U.K.; ⊥ Thomas Young Centre and Department of Materials, Imperial College London, London SW7 2AZ, U.K.; # School of Energy and Chemical Engineering, 131639 Ulsan National Institute of Science and Technology, Ulsan 44919, Republic of Korea

## Abstract

Electrically conductive layered metal–organic frameworks (MOFs) have a wide range of electrochemical applications including in sensors, batteries, spintronics, magnetic semiconductors, and supercapacitors. In these devices, MOF structure strongly influences performance, often through MOF–electrolyte interactions. However, few studies have directly probed these interactions at the electrochemical interface. Recent work showed that ^19^F NMR spectroscopy can probe organic electrolyte environments in the layered MOF Ni_3_(HITP)_2_ (HITP = 2,3,6,7,10,11-hexaiminotriphenylene), revealing that the chemical shifts of in-pore anions are influenced by specific chemical interactions with the MOF functionality. Here, we expand this approach to study ion adsorption in a range of layered MOFs and study the factors influencing the in-pore chemical shifts. We find that all MOF–electrolyte systems display positively shifted in-pore electrolyte resonances, with calculations indicating that both aromatic ring currents and metal-center-induced currents significantly contribute to the observed shifts. We also find that paramagnetic MOFs exhibit additional paramagnetic shifts of the in-pore resonance when specific MOF–electrolyte interactions are present, with paramagnetic NMR calculations linking these to specific coordination geometries and revealing ion binding sites. Finally, MOF particle morphology also strongly affects the appearance of the NMR spectra, with rod-like morphologies leading to slower exchange and better peak resolution. Overall, our results reveal the key factors that influence the NMR spectra of electrolyte sorption in layered MOFs and demonstrate the power of NMR spectroscopy to probe electrochemical interfaces and guide materials design.

## Graphical abstract

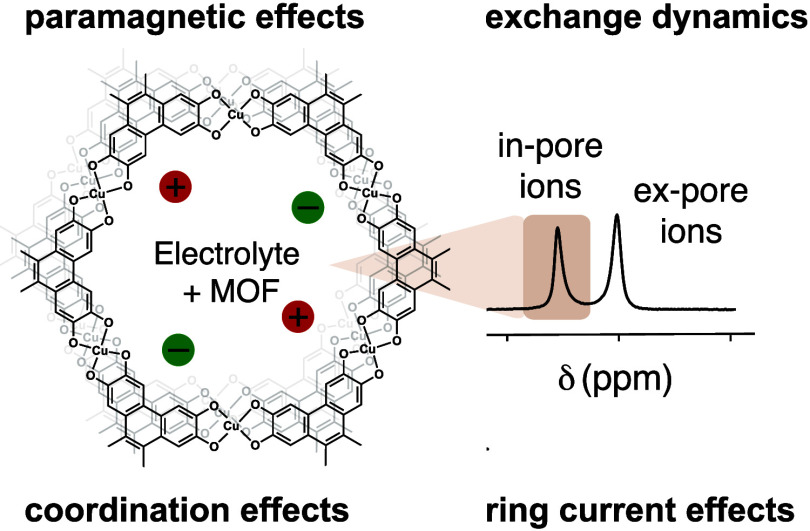

## 1 Introduction

Conductive layered metal–organic frameworks (MOFs) are a class of inorganic materials widely recognized for their intrinsic porosity, conductivity and tunability.^1,2^ As a result of these properties, layered MOFs are attracting growing interest in a number of electrochemical applications, including energy storage devices, electrocatalysis, and sensing.^3–9^ The physical and electronic structures of the layered MOFs are often key to determining device performance. For example, controlling morphology (i.e., MOF crystal size and shape) has been shown to enhance performance in a number of applications, including supercapacitor energy storage devices and electromagnetic wave absorption, with flake-like morphologies (with thin broad crystals) giving rise to higher performances than rod-like morphologies.^10–12^ Moreover, by varying the metal nodes and organic linkers, layered MOFs with different functional groups, pore sizes, and electronic properties can be synthesized, thereby tuning potential interactions between the MOF functionality and the electrolyte. These interactions are believed to play a pivotal role in determining performance across electrochemical devices.^13–15^ However, to fully exploit the tunability of these materials in energy storage devices and other electrochemical applications, a better understanding of how structural variation among MOFs influences MOF–electrolyte interactions, and how these in turn impact performance, is required.

In previous work, we demonstrated that NMR spectroscopy can be used to study specific MOF–electrolyte interactions and electrolyte behavior in electrochemical systems.^16^ We investigated the adsorption of organic electrolytes in Ni_3_(HITP)_2_, primarily using ^19^F NMR spectroscopy of fluorine-containing anions. NMR experiments and calculations showed an increased chemical shift for the in-pore anion resonance relative to the neat electrolyte resonance, and we proposed that specific MOF–anion interactions contributed to this shift and the observed resolution of electrolyte environments in the spectra. Ex situ NMR experiments were also performed to study the charging mechanism of layered MOF supercapacitors. However, as this study was focused on a single MOF, it remains unclear whether these observations can be generalized to other layered MOF–electrolyte systems.

Many questions remain before NMR spectroscopy can be reliably used to understand layered MOF performance in electrochemical devices. These include: (i) Can in-pore electrolyte environments be observed and quantified across different layered MOFs, regardless of functionality, morphology and the presence of paramagnetic metal nodes? (ii) Are specific MOF–electrolyte interactions always the dominant cause of the observed shifts, or do other effects, such as aromatic ring currents and in-pore/ex-pore exchange rates, also play a significant role, as seen in porous carbons? ^17–20^ Ring-current effects, solvation, and coordination have all previously been shown to influence chemical shifts in other MOF systems,^21–25^ while NMR relaxometry has been used to detect adsorbed species in paramagnetic MOFs.^26^ To fully understand the NMR spectra, the contributions of different structural features, such as linker functionality, paramagnetic metal centers, and particle morphology, must be deconvoluted.

This work expands NMR adsorption studies to a wider set of layered MOF–electrolyte systems, enhancing our understanding of factors influencing the NMR spectra of the adsorbed electrolyte. By systematically investigating a range of systems, this study identifies multiple factors that contribute to the observed chemical shifts, including coordination effects, aromatic ring currents, metal-center-induced currents, paramagnetic shifts, and exchange dynamics. Ultimately, the insights from this work pave the way for the broader application of NMR spectroscopy to probe the mechanistic behavior of layered MOFs in a broader range of electrochemical devices

## 2 Results and Discussion

### 2.1 Electrolyte Effects

Our previous work focused on the adsorption of tetrafluoroborate (BF_4_^–^) anions, in the layered MOF nickel­(II) 2,3,6,7,10,11-hexaiminotriphenylene, Ni_3_(HITP)_2_, soaked in 1 M NEt_4_BF_4_ in *d*_3_-acetonitrile (*d*_3_-ACN), using ^19^F NMR spectroscopy (Figure 1a).^16^ The in-pore anion environments were characterized by a positive chemical shift change, Δδ, relative to the neat electrolyte (Δδ = δ_in pore_ – δ_neat electrolyte_). We previously proposed that hydrogen bonding between the charge-dense fluorinated anions (BF_4_^–^, TFSI^–^, and SO_3_CF_3_^–^) and the N–H linker functionality of Ni_3_(HITP)_2_ were responsible for the relatively large Δδ­(^19^F) values observed in NMR spectra (+1.6 to +3.9 ppm; Figure 1a).^16^ Evidence for this included (i) the observation of a larger Δδ value for ^19^F NMR studies of BF_4_^–^ (+3.9 ppm) compared to ^11^B NMR studies (+0.7 ppm), and (ii) the observation of smaller Δδ values for anions with lower charge density on the fluorine atoms (TFSI^–^, SO_3_CF_3_^–^). In contrast to NMR studies of anion adsorption, we could not resolve the in-pore environment for the cations using either ^1^H (NEt_4_^+^) or ^31^P (PEt_4_^+^) NMR spectroscopy, suggesting a low Δδ, potentially due to the absence of favorable interactions between the cations and the MOF functionality. Therefore, to test our previous hypothesis that specific MOF–anion interactions dominate the observed Δδ, here we explore an additional electrolyte, 1 M lithium bis­(trifluoromethylsulfonyl)­imide (LiTFSI) in acetonitrile, in adsorption NMR spectroscopy experiments with Ni_3_(HITP)_2_ (Figure 1b,c). The corresponding Li^+^ cation has a simpler structure, with a smaller ion size and higher charge density than those previously studied. Although LiTFSI has the same anion as a previously studied system and thus was expected to yield similar ^19^F NMR results, the Li^+^ cation differs significantly from previously studied cations. Despite this, no strong specific MOF–electrolyte interactions were anticipated with Li^+^, and poor spectral resolution of the in-pore cation environment was therefore expected.

**Figure 1:**
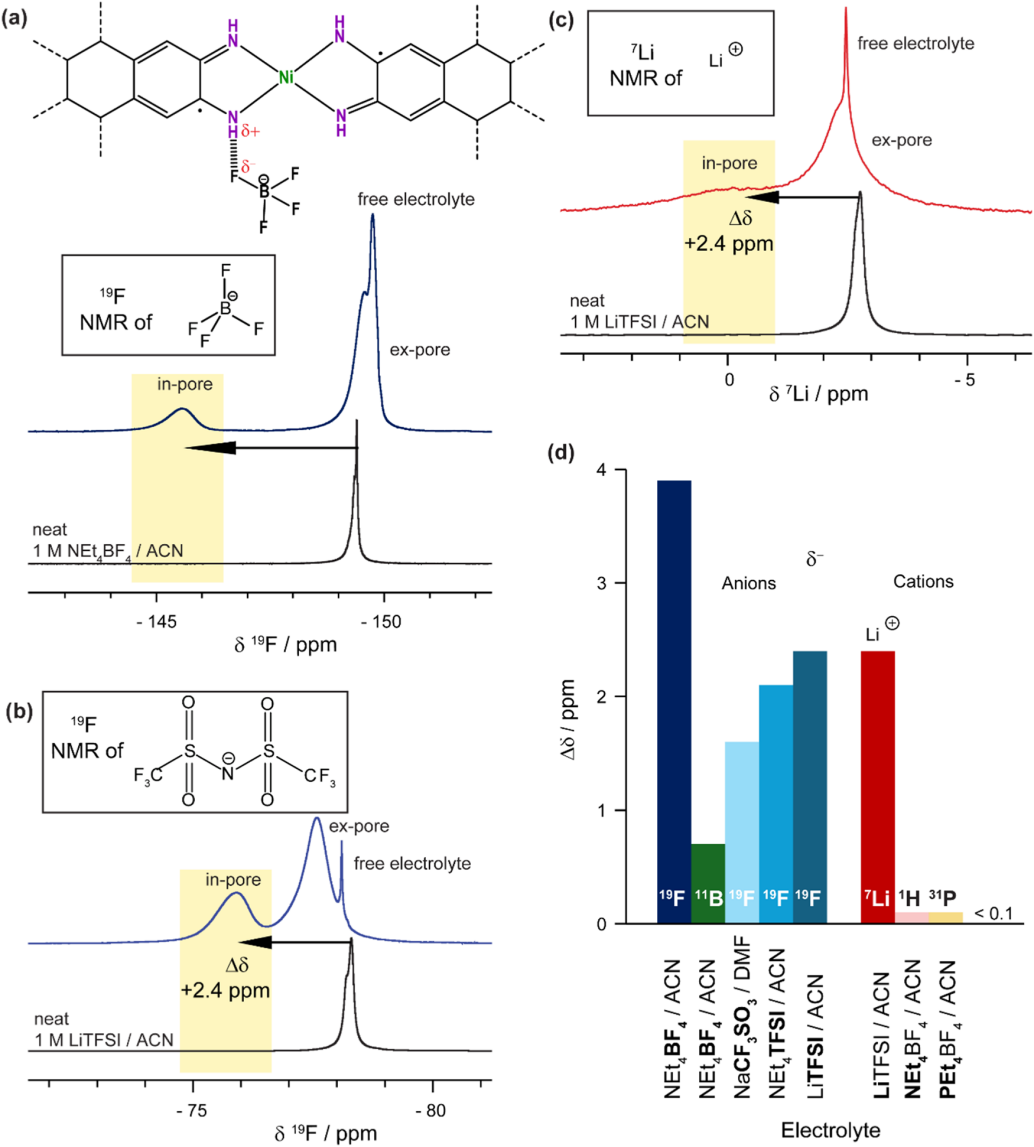
Solid-state quantitative NMR (9.4 T) experiments at 25 kHz magic angle spinning (MAS) of Ni_3_(HITP)_2_ soaked with a saturated loading of (a) 1 M NEt_4_BF_4_/*d*_3_-ACN electrolyte, ^19^F NMR spectrum observing anion environments, with the hypothesized specific MOF–electrolyte interaction with BF_4_^–^ responsible for the large Δδ shown in the associated schematic, (b) 1 M LiTFSI/ACN electrolyte, ^19^F NMR spectrum observing anion environments, and (c) 1 M LiTFSI/ACN electrolyte, ^7^Li NMR spectra observing cation environments. The NMR spectra of the corresponding neat electrolytes are also shown, with some peak asymmetry observed due to imperfect shimming of the magnetic field. The assignments of ion environments are analogous in each case, and both cations and anions display the same Δδ for the in-pore environment with LiTFSI electrolyte. (d) Observed in-pore Δδ against electrolyte, including data from the previously reported study, where the nucleus studied via NMR spectroscopy is indicated.^16^ The measured Δδ for ^1^H NMR and ^31^P NMR was too small to observe in practice.

Ni_3_(HITP)_2_ was synthesized in-house with crystallinity and porosity characterized by powder X-ray diffraction (PXRD) and N_2_ gas sorption at 77 K respectively, with a measured Brunauer–Emmett–Teller specific surface area (BET SSA) of 852 m^2^ g^–1^, in agreement with previous work (SI Figure S1).^16^ For Ni_3_(HITP)_2_ soaked with 1 M LiTFSI/ACN, the in-pore Δδ­(^19^F) for anions was found to be +2.4 ppm (Figure 1b). This is similar to the value previously observed for a TFSI^–^ electrolyte (+2.1 ppm; Figure 1d), and it therefore also can be rationalized as arising from hydrogen-bonding interactions between the anion and the N–H functionality of the linker (Figure 1a). However, unexpectedly, a distinct in-pore cation environment was also revealed by ^7^Li NMR spectroscopy for this MOF–electrolyte system, with a Δδ­(^7^Li) of approximately +2.4 ppm (Figure 1c). This is very similar to the observed Δδ­(^19^F) for the anions and was not anticipated given the absence of strongly negatively polarized adsorption sites on the MOF pore wall that might favor specific Li^+^ binding. Additionally, this finding contrasts with results for other cations (NEt_4_^+^ and PEt_4_^+^), for which no distinct in-pore environments were resolved by either ^1^H or ^31^P NMR spectroscopy with Ni_3_(HITP)_2_ (Figure 1d).^16^ The distinct in-pore shift observed for Li^+^, despite the absence of an expected interaction, suggests that additional factors beyond coordination effects may be contributing to the observed in-pore chemical shifts. These may include aromatic ring current effects (see below), which were not considered significant in our previous study. Notably, the Δδ values observed across all studied MOF–electrolyte systems, while varying in magnitude, consistently show the same sign, supporting the possibility of a common underlying influence such as induced ring currents (Figure 1d). The small size and high charge density of Li^+^ could conceivably allow access to interaction sites inaccessible to larger cations, potentially through intercalation between the MOF layers. Such behavior has been proposed previously for Li^+^ in the nonporous MOF nickel­(II) benzenehexathiolate, Ni_3_(BHT)_2_, by Banda et al., during electrochemical cycling.^27^ However, previous X-ray diffraction studies of Ni_3_(HITP)_2_ and Cu_3_(HHTP)_2_ (HHTP = 2,3,6,7,10,11-hexahydroxytriphenylene) show only very minor structural changes when soaked with electrolyte, with only a ∼1% expansion in the interlayer spacing observed.^15,28^ To explore the origin of these chemical shift contributions further, additional MOF–electrolyte systems were studied.

### 2.2 MOF Functionality Effects

To investigate the relative contributions of specific MOF–electrolyte interactions on the observed in-pore chemical shifts, an almost-isostructural MOF analogue, Zn_3_(HHTP)_2_, was selected (Figure 2a). Both MOFs (Zn_3_(HHTP)_2_ and Ni_3_(HITP)_2_) contain diamagnetic metal nodes, therefore eliminating potential contributions to Δδ from hyperfine shifts of paramagnetic metal centers. Zn_3_(HHTP)_2_ was selected as the linker contains oxygen functionalities (M–O), in contrast to the M–NH groups present in Ni_3_(HITP)_2_. This substitution may favor interactions with cations rather than anions, providing a useful comparison for evaluating ion-specific MOF interactions (Figure 2a). As with Ni_3_(HITP)_2_, Zn_3_(HHTP)_2_ was synthesized reproducibly in-house. Its identity was confirmed by PXRD (SI Figure S2) and porosity was verified by 77 K N_2_ gas sorption isotherms, which displayed Type I profiles with measured BET SSAs of 220–301 m^2^ g^–1^. MOF particle morphology was characterized by scanning electron microscopy (SEM), which revealed rod-like particles similar to those of Ni_3_(HITP)_2_ (SI Figure S3). We note that the surface area is consistently lower than that of Ni_3_(HITP)_2_ (852 m^2^ g^–1^), potentially due to pore blockages from the presence of impurities in the samples (SI Table S2). However, the material was of suitable quality to proceed with NMR adsorption studies.

**Figure 2:**
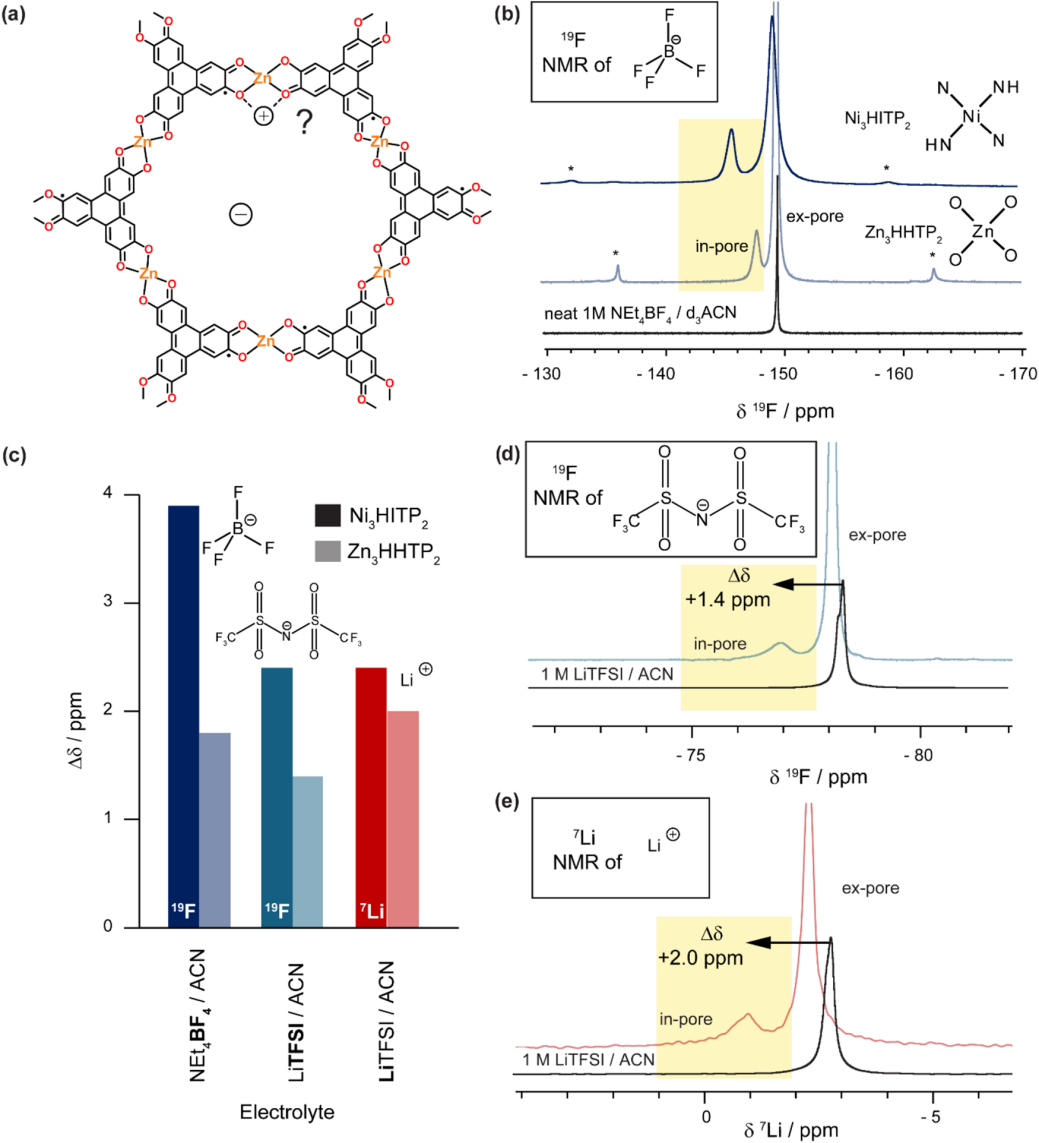
(a) Lewis representation of the hexagonal pore structure of Zn_3_(HHTP)_2_ showing potential interaction sites with cations. The linker is depicted in the anticipated HHTP 3– oxidation state. (b) ^19^F solid-state quantitative NMR (9.4 T) experiments at 5 kHz MAS of Ni_3_(HITP)_2_ Sample A (blue) compared to Zn_3_(HHTP)_2_ Sample A (gray) with a saturated loading of 1 M NEt_4_BF_4_/d_3_ACN electrolyte. The spectra are scaled according to the mass of MOF. The differences in pore functionality are shown. Asterisks (*) indicate spinning sidebands. (c) Observed in-pore Δδ for different electrolytes for Ni_3_(HITP)_2_ (darker) compared with Zn_3_(HHTP)_2_ (paler); the nucleus studied via NMR spectroscopy is indicated. In all cases, the measured Δδ is smaller for Zn_3_(HHTP)_2_. (d, e) Solid-state quantitative NMR (9.4 T) experiments at 25 kHz MAS of Zn_3_(HHTP)_2_ Sample B, soaked with a saturated loading of 1 M LiTFSI/ACN electrolyte, compared with the NMR spectrum of the neat electrolyte (d) ^19^F NMR spectra observing anion environments; (e) ^7^Li NMR spectra observing cation environments. The assignments of ion environments are analogous in each case.

As for Ni_3_(HITP)_2_, we performed electrolyte adsorption studies with Zn_3_(HHTP)_2_ (Figure 2b–d), with an excess of 1 M NEt_4_BF_4_/*d*_3_-ACN added to the MOF. The ^19^F NMR spectrum displayed a lower-intensity peak (−147.6 ppm), positively shifted relative to the neat electrolyte (−149.4 ppm), and a more intense second peak (−149.3 ppm) with a chemical shift closer to that of the neat electrolyte (Figure 2b). These peaks can be assigned to in-pore and ex-pore electrolyte environments in Zn_3_(HHTP)_2_ respectively, analogous to those observed in Ni_3_(HITP)_2_ (Figure 2b). We find that the in-pore integral is lower for Zn_3_(HHTP)_2_ than Ni_3_(HITP)_2_ indicating a lower in-pore anion population (Figure 2b). This is unsurprising given our previous finding that the in-pore population, quantified by the integration of the in-pore resonance, correlates with BET surface area, and that Zn_3_(HHTP)_2_ exhibits a lower BET SSA compared to Ni_3_(HITP)_2_.^16^ The significantly lower Δδ­(^19^F) value for Zn_3_(HHTP)_2_ (+1.8 ppm) compared to Ni_3_(HITP)_2_ (+3.9 ppm; Figure 2c) supports the hypothesis that specific interactions between the anions and the electropositive N–H­(δ+) moieties dominate the in-pore chemical shift in Ni_3_(HITP)_2_, with this contribution absent in Zn_3_(HHTP)_2_ due to the lack of N–H functionalities. The spinning sidebands of the in-pore peak are also no longer more intense than those of the ex-pore environment in Zn_3_(HHTP)_2_, indicating reduced in-pore anisotropy relative to Ni_3_(HITP)_2_.^16^ This may further reflect the absence of strong anion–MOF interactions. In summary, the general form of the ^19^F adsorption NMR spectrum for Zn_3_(HHTP)_2_ mirrors that of Ni_3_(HITP)_2_, confirming that such NMR-based assignments are not exclusive to Ni_3_(HITP)_2_ and can be extended to other layered MOFs.

To further probe the origin of the in-pore Δδ, 1 M LiTFSI in acetonitrile electrolyte was employed (Figure 2d,e). The electronegative M–O functionality on Zn_3_(HHTP)_2_ was anticipated to favorably interact with the Li^+^ cations (Figure 2a). As before, both ^19^F (Figure 2d) and ^7^Li (Figure 2e) NMR spectra revealed two environments, with the in-pore peak positively shifted relative to both the neat electrolyte and the ex-pore peak for both cations and anions. In contrast to Ni_3_(HITP)_2_ (Figure 1), the relative Δδ values for the anions and cations in LiTFSI vary in Zn_3_(HHTP)_2_, with Δδ­(^19^F) = +1.4 ppm and Δδ­(^7^Li) = +2.0 ppm. The slightly greater ^7^Li shift may reflect weak O–Li^+^ interactions. However, the difference is small, and all Δδ values are comparable between the two MOFs, with the exception of BF_4_^–^ in Ni_3_(HITP)_2_ where there is evidence of a strong specific interaction (Figure 2c). Surprisingly, there is no evidence of a larger ^7^Li NMR chemical shift in Zn_3_(HHTP)_2_ relative to Ni_3_(HITP)_2_ due to O–Li^+^ coordination (Figure 2c). Zn_3_(HHTP)_2_ also showed no resolution of an in-pore cation environments with either ^1^H or ^31^P NMR spectroscopy in 1 M PEt_4_BF_4_/ACN (SI Figure S4), consistent with earlier observations for Ni_3_(HITP)_2_.^16^ These results challenge the previously proposed dominance of specific interactions in determining Δδ values in layered MOFs, and suggest that other factors contribute to the observed ^7^Li in-pore chemical shifts in both MOFs. Notably, all in-pore Δδ values measured for Zn_3_(HHTP)_2_ are positive (Figure 2c), consistent with results from Ni_3_(HITP)_2_ (Figure 1d). This suggests a common contribution, such as ring current effects, may be at play. Stronger ring currents in Ni_3_(HITP)_2_ could potentially account for its consistently larger Δδ values.

### 2.3 Ring Current Effects

As the measured in-pore Δδ­(^19^F) and Δδ­(^7^Li) had been consistent in direction and similar in magnitude across many of the MOF–electrolyte systems studied so far (Figure 2c), aromatic ring currents were investigated as a potential contributing factor.^21,29^ The delocalization of π-electrons in aromatic systems induces magnetic fields that alter the local magnetic field at nearby nuclei, giving rise to a nucleus-independent chemical shift (NICS). While ring currents generally produce negative NICS values for adsorbed electrolytes in porous carbons, especially when ions are positioned above the aromatic ring planes, the anisotropy of aromatic ring currents may result in a different NICS effect in layered MOFs, where ions are no longer predominantly located above the linker planes (Figure 2a).^17^

NICS values were calculated for a range of in-plane pore environments in Ni_3_(HITP)_2_ (Figure 3a). All calculated shifts were positive, qualitatively consistent with the experimentally observed positive Δδ values (Figure 1d). The calculated NICS magnitude was approximately +3 ppm, within the range of experimental Δδ values (Figure 3a). This suggests that NICS contributions may therefore be additive to those from specific MOF–electrolyte interactions, helping to explain the largest experimentally measured Δδ values (^19^F Δδ value in BF_4_^–^ with Ni_3_(HITP)_2_; +4 ppm). The relatively small variation in the calculated NICS across in-pore environments, with a reduction of 0.7 ppm at the center of the pore relative to the pore edge, is also consistent with the variation in the observed Δδ for the weakly interacting MOF–electrolyte systems studied in this work (Figure 2c). This implies that even without specific interactions, differences in ion distribution within the pore could result in detectable Δδ differences. However, as the calculated NICS exceeds the measured Δδ in most systems apart from ^19^F NMR of BF_4_^–^ in Ni_3_(HITP)_2_ (Figure 3c), it is likely that the NICS contribution has been slightly overestimated in the calculations. This could arise from limitations in the model, such as the assumption of perfect ring currents in idealized structures, or inherent DFT errors. Moreover, to achieve convergence, the calculations used a doubled interlayer spacing relative to the experimental MOF structure and assumed perfect AA stacking (see Methods). These factors may have further affected the predicted shift values.

**Figure 3:**
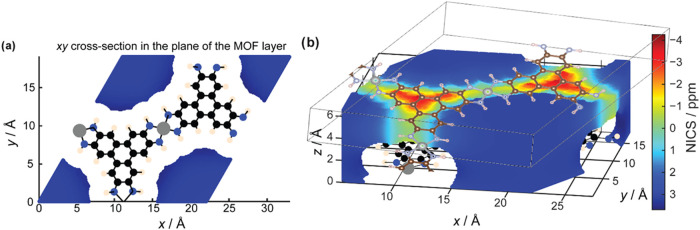
NICS maps calculated for a Ni_3_(HITP)_2_ model with twice the layer spacing as observed in experiments (a) an *xy* cross section, in the plane of the MOF layer, with a positive NICS of around +3 ppm calculated consistently inside the pore (b) between the MOF layers, above the aromatic linker plane, a negative NICS is shown. The calculation cell is shown overlaid.

In contrast to the positive calculated NICS in the MOF pores, a significant negative NICS contribution is predicted in the interlayer regions above and below the linker planes (Figure 3b). Layer offsets in the stacking mode could therefore reduce the local ring current effect at pore edges. In the event of Li^+^ intercalation, Li^+^ cations would be expected to bind above the linker molecules, forming π-cation interactions with the delocalized aromatic electron density.^30^ However, the positive Δδ­(^7^Li) values observed for both MOFs studied strongly suggest that Li^+^ intercalation does not occur, and is therefore unlikely to be the cause of the anomalously high Δδ­(^7^Li) observed for Ni_3_(HITP)_2_. The calculated NICS map for Ni_3_(HITP)_2_ (SI Figure S5) confirms ring currents are present in the aromatic linker and also reveals a significant induced current at the metal center. Both induced currents may therefore contribute to the higher observed shifts in Ni_3_(HITP)_2_ relative to Zn_3_(HHTP)_2_. A weaker net induced-current in Zn_3_(HHTP)_2_ could explain the smaller observed Δδ values in this system, although this needs further investigation.

### 2.4 Paramagnetic Metal Effects

As several recent studies have focused on the application of Cu_3_(HHTP)_2_ as a supercapacitor electrode material, maintaining the same HHTP linker as Zn_3_(HHTP)_2_ but incorporating a paramagnetic Cu­(II) center, we wanted to determine whether our adsorption NMR methodology could be extended to paramagnetic systems, which may be affected by paramagnetic shifts.^11,31,32^ DMF-modulated Cu_3_(HHTP)_2_, which has a rod-like particle morphology, was synthesized to maintain a broadly similar morphology to Ni_3_(HITP)_2_ and Zn_3_(HHTP)_2_.^11^ The MOF identity was confirmed by PXRD, and its porosity was measured by 77 K N_2_ gas sorption isotherms, which displayed Type I behavior with a measured BET SSA of 292 – 346 m^2^ g^–1^, in line with previous reports (314 ± 11 m^2^ g^–1^; SI Figure S6).^11^ After characterization, the MOF was used in NMR adsorption studies and directly compared with Zn_3_(HHTP)_2_ to evaluate the effect of the paramagnetic Cu­(II) center.

As before, Cu_3_(HHTP)_2_ was soaked with 1 M LiTFSI in ACN, and ^7^Li and ^19^F NMR spectra were collected to study the cation and anion environments, respectively (Figure 4). For both cations and anions, three peaks were resolved at saturated loadings, corresponding to in-pore, ex-pore, and free electrolyte, with the in-pore resonances again shifted positively (Figure 4a,b). Strikingly, the in-pore ^7^Li resonance for Cu_3_(HHTP)_2_ (Δδ­(^7^Li) = +5.8 ppm) was significantly more shifted than for Zn_3_(HHTP)_2_ (Δδ­(^7^Li) = +2.0 ppm). Since both MOFs have the same linker functionality (i.e., M–O groups), they would be expected to exhibit similar specific interactions between Li^+^ and the oxygen functionality of the HHTP linker (Figure 4a). While diamagnetic chemical shift calculations on Li^+^ in proximity to a MOF fragment gave reasonably accurate Δδ­(^7^Li) shifts for Zn_3_(HHTP)_2_ (+0.6 ppm), the predicted value for Cu_3_(HHTP)_2_ (−0.5 ppm) is both wrong in sign and an order of magnitude too small (SI Table S3). This discrepancy suggests that the larger Δδ­(^7^Li) value for Cu_3_(HHTP)_2_ is a paramagnetic effect originating from the Cu­(II) center, which was not accounted for in the diamagnetic calculations. In contrast, only a small difference in Δδ­(^19^F) was observed between the two MOFs for the TFSI^–^ anion, where no specific interaction with the MOF functionality is expected (Figure 4b). This suggests that the paramagnetic effect on the chemical shift arises only when there is a specific interaction between the electrolyte ion and the MOF functionality, as is expected between the Li^+^ and the M–O groups in HHTP-based frameworks (Figure 4c).

**Figure 4:**
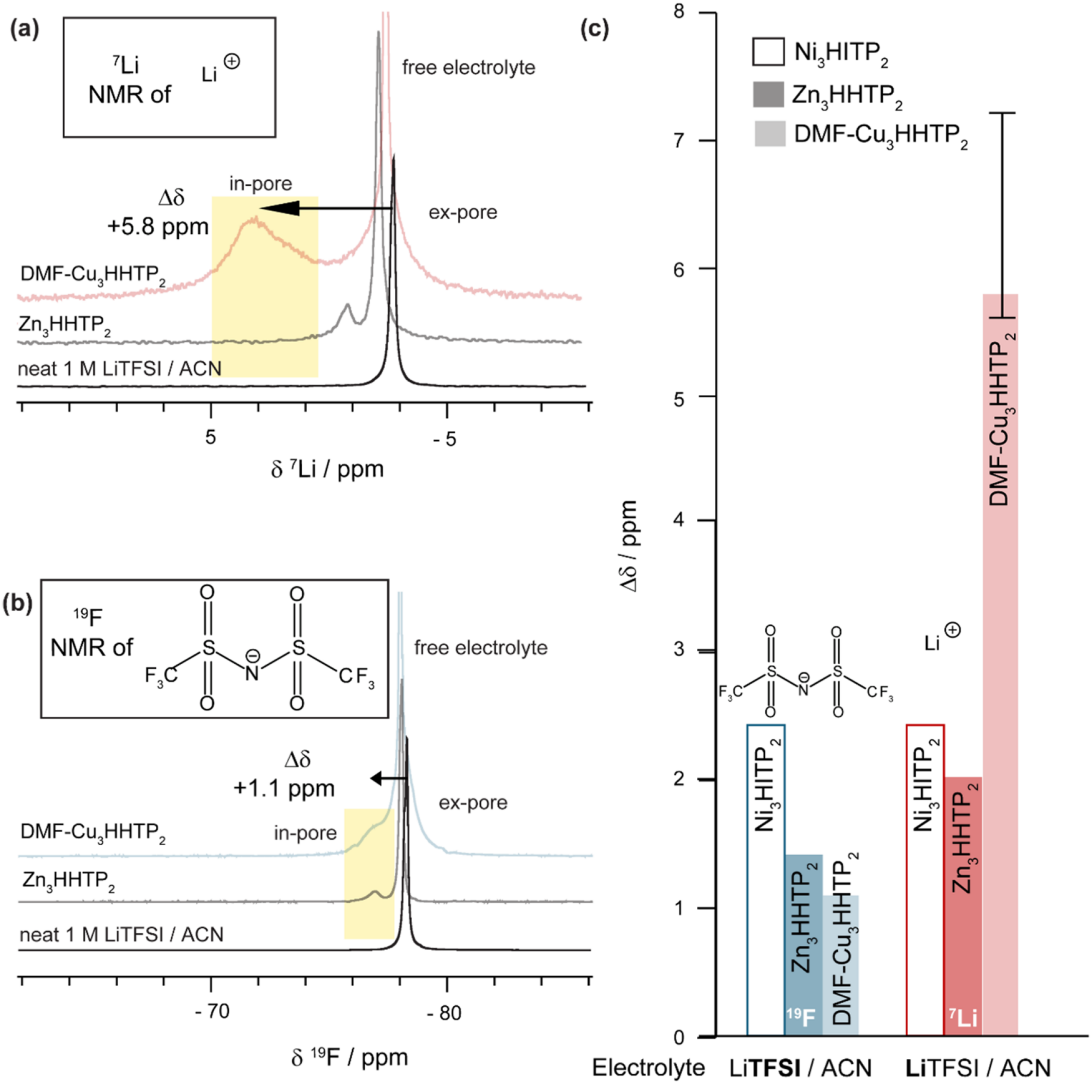
Solid-state quantitative NMR (9.4 T) experiments at 25 kHz MAS of DMF-Cu_3_(HHTP)_2_ Sample A and Zn_3_(HHTP)_2_ Sample B soaked with a saturated loading of 1 M LiTFSI/ACN electrolyte, compared with NMR spectrum of neat electrolyte. Zn_3_(HHTP)_2_ spectra are identical to those in Figure 2. (a) ^7^Li NMR spectra observing cation environments (b) ^19^F NMR spectra observing anion environments. The assignments are analogous in each case. (c) Observed in-pore Δδ­(^19^F) and Δδ­(^7^Li) against electrolyte for Ni_3_(HITP)_2_ (transparent) compared with Zn_3_(HHTP)_2_ (darker) and DMF-Cu_3_(HHTP)_2_ (paler); the nucleus studied via NMR spectroscopy is indicated. Note the anomalously high Δδ­(^7^Li) with DMF-Cu_3_(HHTP)_2_, where the error bar represents the range of results obtained with different MOF batches and electrolyte loadings.

To test the hypothesized paramagnetic influence on the in-pore ^7^Li peak in Cu_3_(HHTP)_2_, variable temperature (VT) NMR experiments were conducted (Figure 5). If the shift arises from a paramagnetic effect, it would be expected to vary with temperature, increasing in magnitude as the temperature decreases, and moving further from the diamagnetic (free electrolyte) region.^33^ Indeed, the in-pore ^7^Li chemical shift increased by +1.8 ppm when the temperature was reduced by 35 °C (Figure 5a). A plot of the inverse in-pore ^7^Li chemical shift (1/δ­(^7^Li)) against temperature gave rise to a strong positive linear correlation (Figure 5b), consistent with Curie–Weiss-type behavior in a high-temperature paramagnetic regime with a positive Weiss constant.^33^ These results confirm that the large in-pore Δδ­(^7^Li) value observed in Cu_3_(HHTP)_2_ is due to a paramagnetic shift induced by the Cu­(II) center, and resulting from the direct interaction between Li^+^ cation and the MOF functionality.

**Figure 5:**
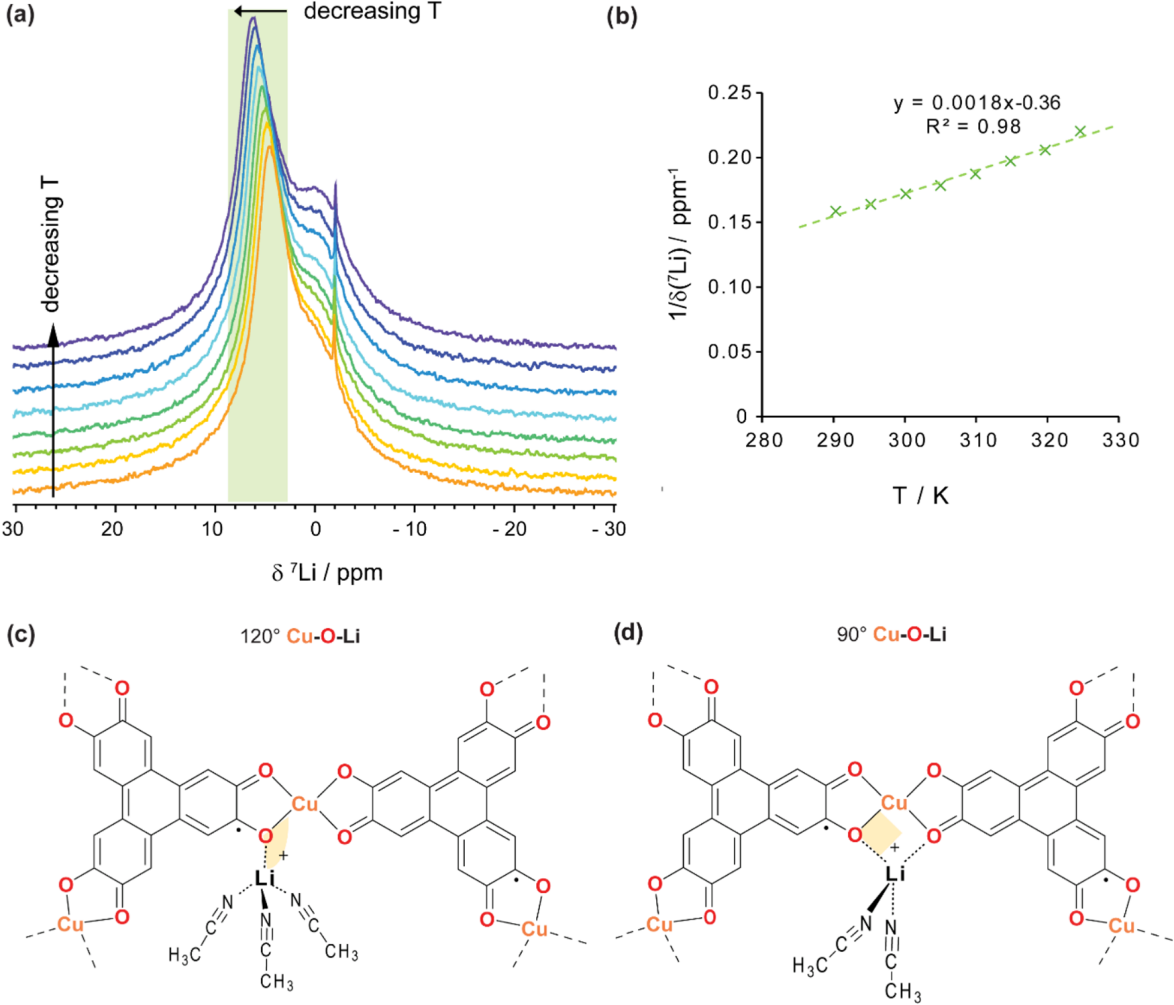
(a) ^7^Li solid-state Variable Temperature (VT) NMR (9.4 T) experiments at 25 kHz MAS of DMF-Cu_3_(HHTP)_2_ Sample B with 1 M LiTFSI in ACN electrolyte. Spectra are decreasing in temperature from bottom (orange, 52 °C) to top (purple, 17 °C). Spectra are recorded with a short recycle delay of 0.1 s to accentuate the paramagnetic shift. The green box highlights the positively shifted in-pore peak, which shifts to higher chemical shifts with decreasing temperature. (b) Inverse chemical shift, δ­(^7^Li), of in-pore peak against temperature. There is a strong positive correlation, with more positive shifts occurring as the temperature decreases. This positive linear trend is indicative of positive spin transfer, with an estimated Weiss constant of 420 K.^33^ (c, d) Simulated geometries of Li^+^ coordinated to Cu_3_(HHTP)_2_ fragment (c) Cu–O–Li in approximately a 120° configuration, where Li^+^ is coordinated by one oxygen pathway and solvated by three acetonitrile molecules. (d) Cu–O–Li in approximately a 90° configuration, where Li^+^ is coordinated to two oxygen pathways and solvated by two acetonitrile molecules. The overall coordination geometry for both environments is a distorted tetrahedron. The linker is depicted in the anticipated HHTP 3– oxidation state.

To understand the local environments responsible for the paramagnetic ^7^Li NMR shifts seen in Cu_3_(HHTP)_2_, paramagnetic NMR simulations were performed for Li^+^ in two different coordination geometries relative to the Cu­(II) node (Figure 5c,d). In the first geometry, Li^+^ was optimized with three solvating acetonitrile molecules, resulting in a distorted tetrahedral coordination configuration around Li^+^, and a Cu–O–Li pathway with an angle of 119.1° (Figure 5c). The resulting simulated paramagnetic shift was +9 ppm, closely matching the experimentally observed Δδ­(^7^Li) range of +5.8–7.6 ppm in VT NMR experiments (Figure 5a). Therefore, we assign the positively shifted in-pore environment predominantly to Li^+^ cations in this 120° Cu–O–Li coordination geometry. Such pathways are expected to give rise to small Fermi-contact shifts due to competing spin-delocalization mechanisms between ideal 90 and 180° configurations and reduced orbital overlap. Fermi-contact shifts may occur in either direction and are challenging to predict without simulation.^34–36^ Similar small positive shifts have been reported for Li^+^ ions in lithium transition metal oxides.^36^

In the second geometry, Li^+^ was optimized with two solvating acetonitrile molecules in the presence of a single MOF layer, forming a ring coordination with two linker oxygen atoms and completing an approximate square with the Cu^2+^ node (Figure 5c). This geometry forms two 91.6° Cu–O–Li pathways and gave rise to a simulated negative Fermi-contact shift of −34 ppm. This shift is consistent with the Goodenough–Kanamori rules,^37,38^ where Li^+^ coordination at ∼90° to a metal (Cu) center, with a configuration resembling filled t_2g_ orbitals in octahedral symmetry, and two orthogonal oxygen orbitals results in strong spin transfer and a negative shift contribution.^37,39–41^ Although this predicted chemical shift differs substantially from that observed in the main adsorption NMR experiments, our recent ex situ NMR experiments on supercapacitors using the same MOF–electrolyte system revealed this environment under negative charging conditions, indicating that the preferred Li^+^ coordination geometry can change depending on the electrochemical state.^15^ Furthermore, adsorption experiments on DMF-Cu_3_(HHTP)_2_ at higher temperatures showed a similarly negatively shifted ^7^Li peak at −27 ppm, with partial reversibility upon cooling (SI Figure S7). The interchangeability of these environments demonstrate that they are intrinsically connected, with redistribution of coordination geometries possible. The observation of these two paramagnetic environments in Cu_3_(HHTP)_2_ is therefore not exclusive to charged samples.

In summary, paramagnetic metal centers in layered MOFs can induce large in-pore Δδ­(^7^Li) values through Fermi-contact shifts, provided there is specific coordination between the electrolyte ion and the M–O functionalities. The unique ability of NMR spectroscopy to directly observe these local coordination sides highlights its value in identifying performance-enhancing specific interactions in various electrochemical systems.^15^

### 2.5 MOF Particle Morphology Effects

In previous work, subtle differences in particle morphology between Ni_3_(HITP)_2_ samples were proposed to influence the appearance of the NMR spectrum.^16^ Having characterized the electrolyte environments in rod-like DMF-modulated Cu_3_(HHTP)_2_, we investigated the impact of particle morphology on NMR adsorption spectra by synthesizing Cu_3_(HHTP)_2_ with a different morphology. Indeed, the morphology of this MOF can readily be tuned via coordination modulation during the synthesis.^11^ While the DMF-modulated synthesis produces MOF particles with a rod morphology (diameter 110–340 nm, length 0.5–4 μm), NH_3_-modulated synthesis produces MOF particles with a flake-like morphology (diameter 0.5–2.9 μm, thickness 30–110 nm; Figure 6a).^11^ The flake-like Cu_3_(HHTP)_2_ particles were synthesized and then characterized via PXRD (SI Figure S6a) and 77 K N_2_ gas sorption (SI Figure 6b). All samples exhibited good crystallinity and porosity, with BET surface areas ranging from 778–875 m^2^ g^–1^, in agreement with literature values (759 ± 32 m^2^ g^–1^).^11^ While we note that the most striking difference between the flake- and rod-Cu_3_(HHTP)_2_ samples is their particle morphology, we note that the two samples also have differences in framework layer-stacking (SI Figure S6a).

**Figure 6:**
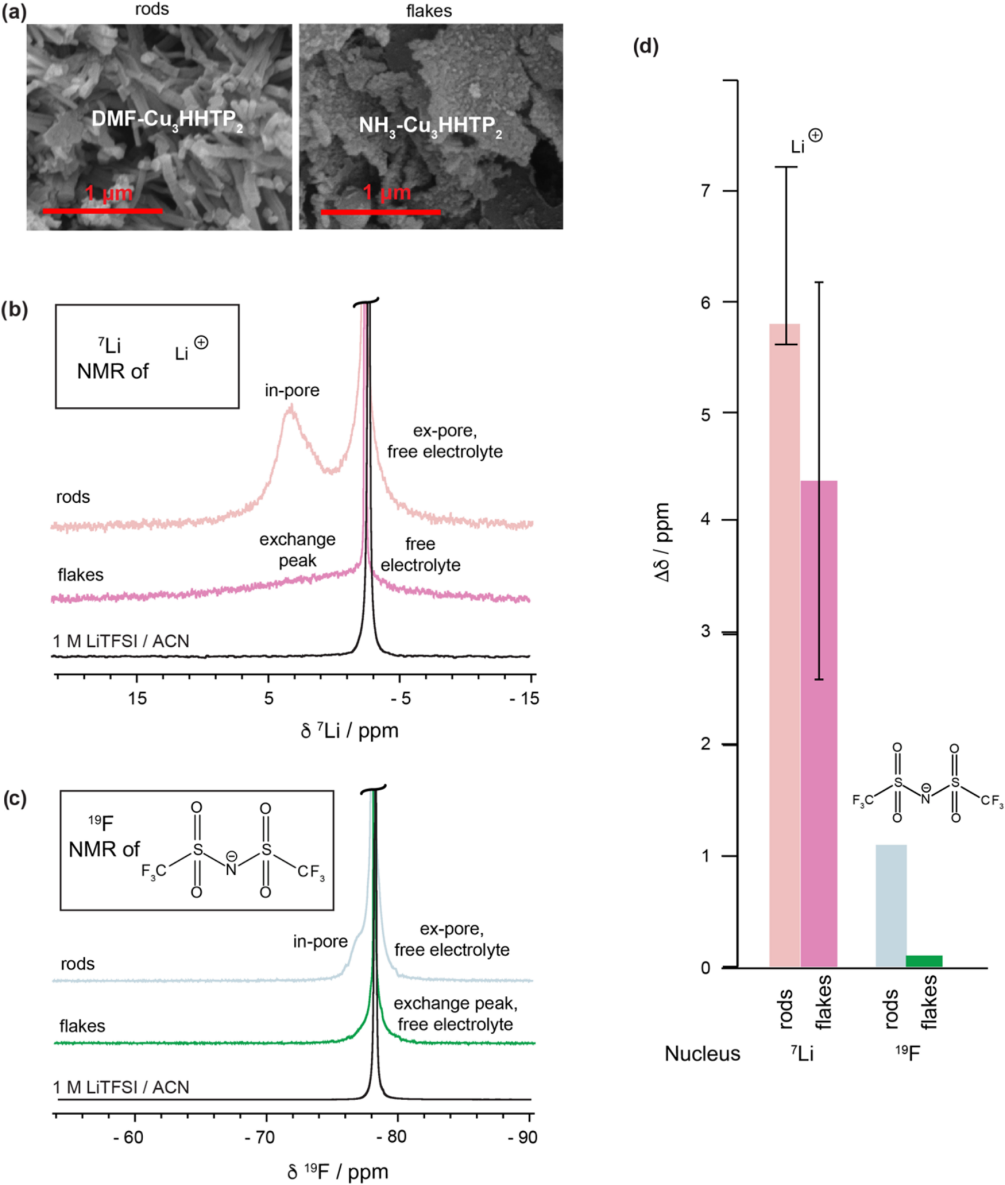
(a) Scanning electron microscopy (SEM) images of selected samples of DMF-Cu_3_(HHTP)_2_, with rod-like morphology, and NH_3_–Cu_3_(HHTP)_2_, showing flake-like morphology. (b, c) Solid-state quantitative NMR (9.4 T) experiments at 25 kHz MAS of DMF-Cu_3_(HHTP)_2_ rods Sample A and NH_3_–Cu_3_(HHTP)_2_ flakes, soaked with a saturated loading of 1 M LiTFSI/ACN electrolyte, compared with NMR spectrum of neat electrolyte. See Figure S8 for spectral fits. The DMF-Cu_3_(HHTP)_2_ rods NMR spectra are identical to those shown in Figure 4. (b) ^7^Li NMR spectra observing cation environments (c) ^19^F NMR spectra observing anion environments. The assignments are analogous in each case, where the broader resonance for NH_3_–Cu_3_(HHTP)_2_ flakes likely represents an intermediate exchange regime. (d) Observed in-pore Δδ against electrolyte for DMF-Cu_3_(HHTP)_2_ rods compared with NH_3_–Cu_3_(HHTP)_2_ flakes; the nucleus studied via NMR spectroscopy is indicated. The bar represents the measured ‘in-pore’ Δδ at a saturated loading, and the error bar represents significant variation in fitted Δδ value with MOF batch and electrolyte loading. The measured Δδ for NH_3_–Cu_3_(HHTP)_2_ flakes is likely lower due to a faster exchange resulting in a weighted average recorded for the in-pore chemical shift.

Following this, Cu_3_(HHTP)_2_ flakes were used in NMR adsorption experiments with 1 M LiTFSI/ACN electrolyte (Figure 6b,c). Since both forms of Cu_3_(HHTP)_2_ share very similar chemical structures, differences in their NMR spectra are expected to arise primarily from differences particle morphology, although we cannot rule out minor contributions from differences in the layer stacking of these samples. In both the ^7^Li (Figure 6b) and ^19^F (Figure 6c) NMR spectra, the well-resolved in-pore peak observed for the rod-like MOF is no longer present in the NH_3_-modulated Cu_3_(HHTP)_2_ flakes. Instead, the spectra can be fitted with a broad resonance, slightly positively shifted from the neat electrolyte, and a sharp resonance, for which the line shape resembles that of the free electrolyte rather than a typical ex-pore environment (SI Figure S8). This reduction in the number of resolved environments suggests that the in-pore and ex-pore species are now in intermediate or fast exchange on the NMR time scale, while still exchanging more slowly with isolated pockets of free electrolyte. The sensitivity of this system to the intermediate exchange regime may also explain the high observed variability in chemical shift and line shape for the in-pore resonance in Cu_3_(HHTP)_2_ flakes observed as the electrolyte loading and the sample batch are varied (Figure 6d and SI Figure S9). This shift in exchange behavior between the two samples of Cu_3_(HHTP)_2_ is consistent with their structural differences. Due to their long, narrow channels and limited pore openings, Cu_3_(HHTP)_2_ rods are expected to exhibit slower ion exchange than flakes, which have a shorter pore length and more openings that promote faster exchange.

This exchange hypothesis was further tested through: (i) Exchange spectroscopy (EXSY), to probe slow exchange in Cu_3_(HHTP)_2_ rods (Figure 7a), and (ii) VT NMR, to investigate the proposed intermediate exchange in Cu_3_(HHTP)_2_ flakes (Figure 7b). ^7^Li EXSY experiments performed on Cu_3_(HHTP)_2_ rods soaked with 1 M LiTFSI/ACN electrolyte showed clear in-pore/ex-pore cross peaks with a mixing time of 23 ms, indicative of slow exchange on the NMR time scale between the environments, and consistent with expectations for this morphology (Figure 7a and SI Figure S10). In contrast, much faster exchange was confirmed for Cu_3_(HHTP)_2_ flakes by ^7^Li VT NMR, where both the in-pore and ex-pore environments became distinguishable only at decreasing temperatures (Figure 7b). These observations support a faster rate of exchange in NH_3_–Cu_3_(HHTP)_2_ flakes than in DMF-Cu_3_(HHTP)_2_ rods. This higher exchange rate is likely responsible for the reduced resolution of in-pore and ex-pore environments in the flake-like morphology (see SI for further discussion).

**Figure 7:**
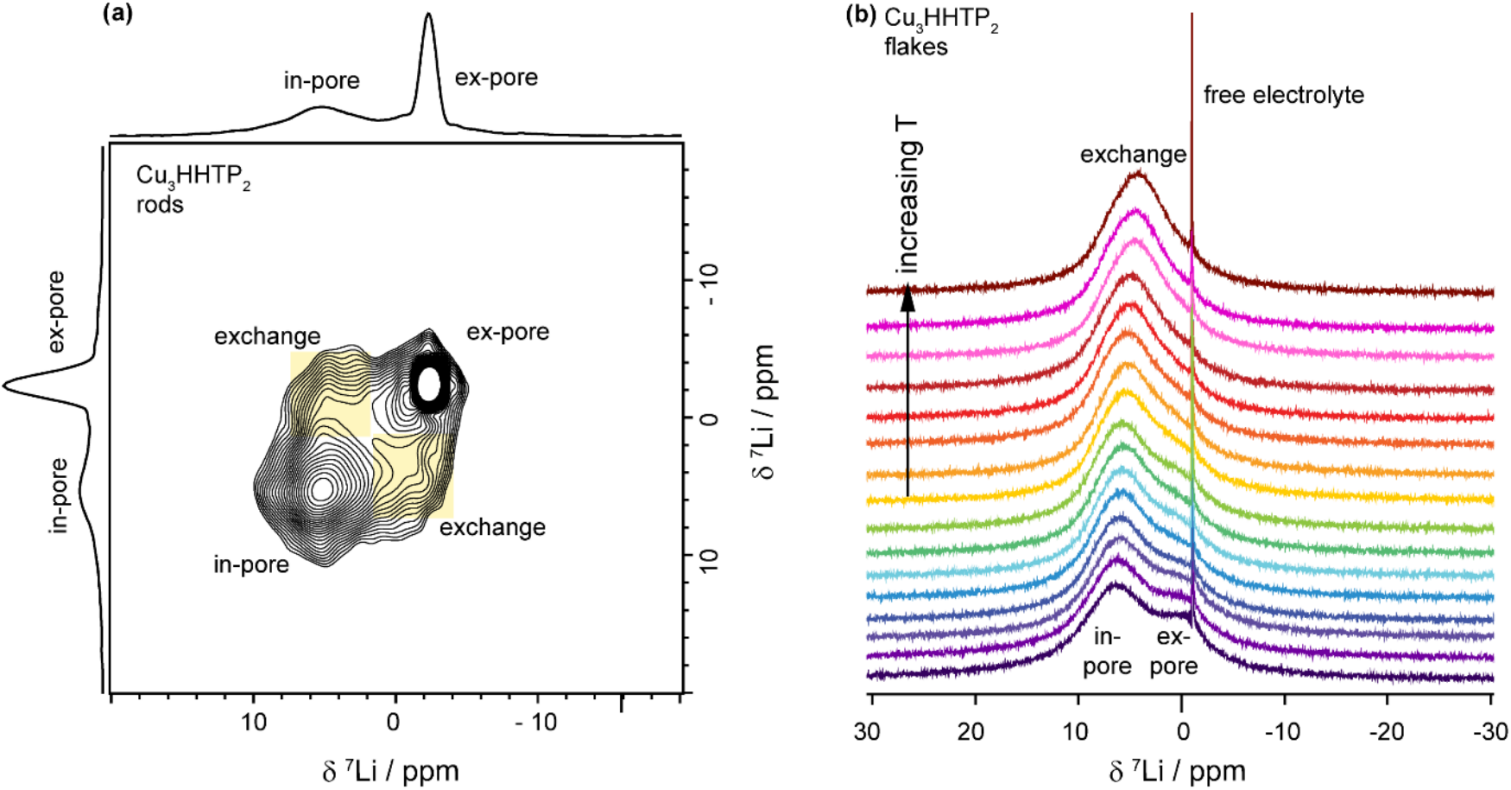
(a) ^7^Li solid-state EXSY NMR (9.4 T) experiments at 25 kHz MAS of DMF-Cu_3_(HHTP)_2_ rods Sample B with 1 M LiTFSI in ACN electrolyte, with a mixing time of 23 ms. Highlighted yellow regions indicate exchange cross peaks. (b) ^7^Li solid-state VT NMR (9.4 T) experiments at 25 kHz MAS of NH_3_–Cu_3_(HHTP)_2_ flakes Sample A with 1 M LiTFSI in ACN electrolyte. Spectra are increasing in temperature from bottom (purple, 17 °C) to top (brown, 64 °C).

The appearance and resolution of in-pore environments across different MOFs studied in this work can be rationalized by their similar morphologies, with both Ni_3_(HITP)_2_ and Zn_3_(HHTP)_2_, like DMF-Cu_3_(HHTP)_2_, also exhibiting rod-like particle morphologies (SI Figure S3). Future NMR studies of Ni_3_(HITP)_2_ and Zn_3_(HHTP)_2_ samples with varied morphologies (when available) could provide further support for our observations for Cu_3_(HHTP)_2_. Ultimately, electrolyte adsorption NMR experiments have therefore been found to be highly sensitive to sample morphology, with the best resolution of environments achieved in slow exchange regimes typical of rod-like morphologies.

Although MOFs with flake-like morphologies often have superior electrochemical performance, their dynamic behavior complicates detailed characterization by NMR.^10,11^ The rapid exchange processes observed in these materials can obscure the ability to resolve and quantify in-pore species, as demonstrated by the limitations encountered with Cu_3_(HHTP)_2_ flakes. However, careful experimental design, such as going to lower temperatures or using higher magnetic field strengths, could effectively mitigate these effects and provide valuable insights into MOF–electrolyte specific interactions. Where high spectral resolution cannot be achieved, direct comparisons between MOFs of different morphologies should be avoided in electrolyte adsorption NMR studies.

## 3 Conclusions

This work has demonstrated that NMR spectroscopy is a powerful technique for characterizing electrolyte environments in a range of layered MOF systems, including both Zn_3_(HHTP)_2_ and Cu_3_(HHTP)_2_. We show that the observed chemical shifts of in-pore cationic and anionic species depend not only on specific MOF–electrolyte interactions, but also on factors such as aromatic ring currents, induced currents at the metal center, paramagnetism, MOF particle morphology, and ion exchange dynamics.

Notably, all MOF-electrolyte systems exhibited positive in-pore Δδ values. Aromatic ring currents were investigated as a potential cause of these consistently positive values via NICS simulations. These simulations reproduced the experimental trend in sign but overestimated the magnitude, likely due to structural defects or limitations of the DFT model used. Metal-center-induced currents were also revealed as a potential source of chemical shift variation between MOFs.

In Cu_3_(HHTP)_2_, the paramagnetic Cu­(II) nodes further influenced the in-pore chemical shift when specific interactions with Li^+^ were present. VT NMR confirmed a temperature-dependent paramagnetic contribution, and simulations indicated a Cu–O–Li coordination geometry of ∼120°, supporting the assignment of this environment to paramagnetic interactions. This demonstrates the applicability of NMR spectroscopy for probing specific interaction sites in layered MOFs.

Morphology was also found to strongly influence spectral resolution due to exchange dynamics. ^7^Li EXSY of rod-like Cu_3_(HHTP)_2_ revealed slow exchange between in-pore and ex-pore environments, while VT NMR of flake-like Cu_3_(HHTP)_2_ confirmed faster exchange regimes. Fast ion exchange in flake-like systems ultimately limits the resolution and quantification of distinct electrolyte environments. Using lower temperatures or higher magnetic field strengths may help to improve spectral resolution, enabling clearer insights into MOF–electrolyte interactions.

Overall, this study highlights the broad applicability of NMR spectroscopy in probing complex MOF–electrolyte systems. Factors such as specific interactions, aromatic ring currents and metal-center-induced currents, paramagnetism, and particle morphology were all found to influence the spectra. While the scope of our study was limited to layered MOFs, the previously mentioned factors will also influence the chemical shifts for molecules adsorbed in MOFs more generally, including those with nonlayered structures. Future studies should aim to quantitatively deconvolute these effects and extend our methodology to new frameworks and electrochemical devices.

## Supplementary Material



## Data Availability

All raw experimental data files are available in the Cambridge Research Repository, Apollo, with the identifier DOI: 10.17863/CAM.119811

## Notes

Experimental details, detailed discussion of analysis, figures (PDF)
