# Supplementary material for: Factors Impacting the Nuclear Magnetic Resonance Spectra of Electrolyte Adsorbed in Layered Metal–Organic Frameworks
Source: J Phys Chem C Nanomater Interfaces. 2025 Nov 19;129(47):21035–46. doi: 10.1021/acs.jpcc.5c04963 (PMC12670500; doi:10.1021/acs.jpcc.5c04963)
Supplement: Supplementary file 1 [file jp5c04963_si_001.pdf]

Supplementary Information for:

Factors Impacting the Nuclear Magnetic Resonance Spectra of Electrolyte Adsorbed in Layered Metal-Organic Frameworks

Chloe J. Balhatchet[1], Jamie W. Gittins[1], Ieuan Seymour[2], Ryan Bragg[3,4], Seung-Jae Shin[5,6], Teedhat Trisukhon[1], Thomas Kress[1], John M. Griffin[3], Alexander C. Forse[1*]

[1] Yusuf Hamied Department of Chemistry, University of Cambridge, Lensfield Road, Cambridge CB2 1EW, UK.

[2] Department of Chemistry, University of Lancaster, Lancaster, LA1 4YW, UK.

[3] Advanced Centre for Energy and Sustainability (ACES), Department of Chemistry, University of Aberdeen, Meston Walk, Aberdeen, AB24 3UE, UK.

[4] Department of Chemistry, University of Warwick, Coventry, CV4 7AL, UK.

[5] Thomas Young Centre and Department of Materials, Imperial College London, London SW7 2AZ, UK.

[6] UNIST, 50, UNIST-gil, Ulsan 44919, Republic of Korea.

[*]Corresponding author's email: acf50@cam.ac.uk

Table of Contents

Synthesis 1
Materials Characterisation **2**
X-Ray Diffraction 2
Gas Sorption 2
Scanning Electron Microscopy 2
Elemental Analysis 2
Adsorption NMR Sample Preparation **2**
NMR Experiments **3**
Ring-Current Calculations **3**
QM/MM Simulation Details **4**
Paramagnetic Simulations **4**
SI Figure S1 .... **6**
SI Figure S2 .... **6**
SI Figure S3 .... **7**
SI Table S2 .... **7**
SI Figure S4 .... **8**
SI Figure S5 .... **9**
SI Figure S6 .... **10**
SI Table S3 .... **10**
SI Figure S7 .... **11**
SI Figure S8 .... **12**
SI Figure S9 .... **13**
SI Figure S10 .... **14**
SI Figure S10 .... **15**
Further Discussion on Exchange **16**
References **17**

Synthesis

$Ni_3(HITP)_2$ was synthesised as described in our previous publication.[1] A solution of 323 mg of $NiCl_2.6H_2O$ (1.36 mmol, 1.5 eq) in 20 mL of water was added to a solution of 487 mg (0.91 mmol, 1 eq) of HATP.6HCl in 140 mL of water. To this was added 3.5 – 4.5 mL of concentrated aqueous ammonia (18 M). The resulting mixture was heated to 60 °C in an oil bath and stirred for 2 h with air bubbling through a needle. The resulting crude black precipitate was separated from the reaction mixture by centrifugation and washed with deionized water (8 × 135 mL) and ethanol (3 × 135 mL). Finally, the solid product was dried under dynamic vacuum on a Schlenk line at 85 °C for a minimum of 48 h. The product was kept in a nitrogen-filled glovebox until used.

$Zn_3(HHTP)_2$ was synthesised by a modified literature procedure.[2] Zinc acetylacetonate $Zn(C_5H_7O_2)_2$ (0.159 g, 0.587 mol, 1.72 eq.) and H_6HHTP, (0.111 g, 0.342 mmol, 1.00 eq.) were suspended in distilled water (15 mL), and the solution stirred for 15 mins. N-Methyl-2-pyrrolidone, NMP (1.5 mL, 46 eq.) was added, and the mixture was sonicated for 30 mins. The resulting mixture was heated in an oven for 12 h at 80 °C and allowed to cool for 1.5 h. The resulting crude black precipitate was separated from the reaction mixture by centrifugation and washed with deionised water (3 × 135 mL), ethanol (3 × 135 mL), and acetone (3 × 135 mL). Finally, the solid product was dried under dynamic vacuum on a schlenk line at 80 °C for a minimum of 72 h. The product was kept in a nitrogen-filled glovebox until used.

DMF-modulated $Cu_3(HHTP)_2$ was synthesised according to a modified literature procedure.[3] Copper(II) sulfate, $Cu(SO_4)_2.5H_2O$ (0.205 g, 0.820 mmol, 2.27 eq.), was dissolved in distilled water (9 mL). A second solution of H_6HHTP, (0.117 g, 0.361 mmol, 1.00 eq.) in distilled water (12 mL) and DMF (1.5 mL, 19.5 mmol, 54.0 eq.) was prepared, and sonicated (15 mins). The resulting solutions were heated in an oven at 80 °C for 10 mins, then mixed and heated further at 80 °C for 12 h and allowed to cool for 1.5 h. The resulting crude black precipitate was separated from the reaction mixture by centrifugation and washed with deionised water (3 × 135 mL), ethanol (3 × 135 mL), and acetone (3 × 135 mL). Finally, the solid product was dried under dynamic vacuum at 75 °C in a vacuum oven for a minimum of 72 h. The product was kept in a nitrogen-filled glovebox until used.

NH_3-modulated $Cu_3(HHTP)_2$ was synthesised according to the literature procedure.[4] Copper(II) nitrate, $Cu(NO_3)_2.3H_2O$ (0.127 g, 0.526 mmol, 1.65 eq.), was added to distilled water (2 mL) and ammonia solution, 35 %, (0.829 mL, 15.0 mmol, 47 eq.) to produce a dark blue solution. H_6HHTP, (0.103 g, 0.318 mmol, 1.00 eq.) was dispersed in distilled water (8.5 mL), and the blue copper solution added dropwise to the dispersion. The resulting mixture was heated in an oven at 80 °C for 24 h and allowed to cool for 1.5 h. The resulting crude black

precipitate was separated from the reaction mixture by centrifugation and washed with deionised water (3 × 135 mL), ethanol (3 × 135 mL), and acetone (3 × 135 mL). Finally, the solid product was dried under dynamic vacuum at 75 °C in a vacuum oven for a minimum of 72 h. The product was kept in a nitrogen-filled glovebox until used.

Materials Characterisation

X-Ray Diffraction

Powder X-Ray Diffraction data were obtained in-house using a Malvern Panalytical Empyrean instrument with non-chromated CuK_α radiation (λ = 1.5406 Å) and a X'celerator Scientific detector. Samples were placed in a glass sample holder and measurements were performed at room temperature with a θ/2θ Bragg-Benato geometry. A divergence slit of 1/8 inch was found to be optimal, and a 2θ range of 3° to 50° with a step size of 2θ = 0.017° was used, with a resulting experiment time of 1 h. Simulated PXRD were produced using VESTA version 4.6.0.[5]

Gas Sorption

An Anton Parr Autosorb iQ-XR was used to obtain all low pressure N_2 isotherms at 77 K. *Ex-situ* degassing (90 – 120 °C, 16 h) was performed for all samples.

AsiQwin version 5.21 software was used for evaluation of sorption isotherms and the calculation of BET areas using Rouquerol's consistency criteria.[6] All samples exhibited Type I N_2 isotherms, indicative of microporosity in the MOFs.

Scanning Electron Microscopy

MOF samples were mounted onto a stainless-steel scanning electron microscopy (SEM) stub. A Quorum Technologies Q150T ES Turbo-Pumped Sputter Coater was used to sputter-coat the samples with 10 nm Pt to improve the conductivity for imaging. Imaging was performed on a Tescan MIRA3 FEG-SEM, with a 5 keV beam voltage and working distance of 7 – 8 mm.

Elemental Analysis

Inductively Coupled Plasma Optical Emission Spectroscopy (ICP-OES) using a Thermo Scientific iCAP-7400 ICP spectrometer was used to determine Zn content of $Zn_3(HHTP)_2$. CHN combustion analysis (975 °C) using an Exeter Analytical CE-440 was used for C, H, and N content.

Adsorption Sample Preparation for NMR Experiments

For adsorption sample preparation, activated MOF powder was packed into zirconia magic-angle spinning (MAS) rotors (2.5 mm outer diameter) in an N_2-glovebox. The rotor was

weighed before and after adding the MOF material, and then organic electrolyte was added with a micro-syringe to saturate the powder sample, and the capped rotor was weighed again. Samples were left overnight (> 16 h) for complete soaking before starting NMR experiments.

Solid-State NMR Experiments

Solid-state NMR experiments were performed on a Bruker Avance Neo spectrometer with magnetic field strength of 9.4 T, corresponding to ^{19}F and ^{7}Li Larmor frequencies of 376.7 MHz and 155.6 MHz. All NMR experiments were performed using a Bruker 2.5 mm double-resonance MAS probe at room temperature, unless otherwise specified. The temperature was calibrated in VT NMR experiments using an inversion-recovery ^{79}Br pulse sequence applied to KBr (Figure S11).[7] All spectra were performed at an MAS rate of 5 or 25 kHz as necessary to achieve satisfactory resolution.

The range of radiofrequency field (RF) strengths and quantitative recycle delays used for each nucleus is given in **Table S1**. The 90 ° pulse length was optimised on every sample.

Nucleus Studied	RF Strength / kHz	Quantitative Recycle Delay / s
^{7}Li	71-100	0.1-4
^{19}F	59-125	4-12

Table S1: Range of RF strengths and quantitative recycle delays found for all NMR experiments in this work.

^{7}Li chemical shifts were referenced externally to lithium chloride at −1.0 ppm and ^{19}F chemical shifts to hexafluorobenzene at −164.9 ppm. All spectra are normalised according to the number of scans performed; spectra on the same figure are further normalised to the mass of MOF in the sample. Fitting of spectra was performed using dmfit software using a Lorentzian line shape for all peaks and a chemical shift anisotropy model (Haeberlen convention).[8]

Ring-Current Calculations

Periodic electronic structure calculations for $Ni_3(HITP)_2$ layers were performed using first-principles plane-wave density functional theory (DFT), as implemented in CASTEP v22.11. The Perdew-Burke-Erzernhof (PBE)[9,10] nonhybrid generalised gradient approximation (GGA) functional was employed, along with a plane-wave basis energy cut-off of 700 eV and ultrasoft pseudopotentials. The Grimme-D3 dispersion correction scheme was applied for all calculations.[11] NMR shielding tensors were computed using the gauge-including projector augmented wave (GIPAW) approach.[12,13] Structural optimisations were carried out with the full relaxation of atomic positions and lattice parameters, subject to convergence tolerances of 0.0005 eV, 0.03 eV Å^{-1}, and 0.001 Å for the energy, force and displacement, respectively. During optimisation, interlayer distances were constrained by fixing the lattice parameter

perpendicular to the $Ni_3(HITP)_2$ layer. To ensure the convergence of magnetic shielding parameters with respect to *k*-space sampling, interlayer distances were set at twice the experimentally derived values (6.516 Å) to reduce the metallic character of the system. Two-dimensional Monkhorst-Pack *k*-grids (*k* x *k* x 1) oriented parallel to the $Ni_3(HITP)_2$ layer provided sufficiently dense sampling to accurately describe the electronic structure. Shielding contributions from macroscopic susceptibility effects were omitted.

QM/MM Simulation Details

QM/MM simulations were conducted to investigate LiTFSI–MOF interfaces, with parameters taken from previous work.[14]

The chemical shift is calculated using NWChem software[15] with the Gauge Including Atomic Orbitals (GIAO) formalism[16]. The calculated isotropic chemical shift, σ is scaled to compute the chemical shift, δ, using the following the equation:

$$\delta = \frac{\text{intercept} - \sigma}{-\text{slope}} \tag{S1}$$

where the intercept and slope values were taken from previous work.[14] The input geometries were selected using equilibrated QM/MM structures including Li^+ and its first solvation shell. In case of adsorbed Li^+, a $Zn_3(HHTP)_2$ or $Cu_3(HHTP)_2$ fragment is also included. The geometry optimisation is conducted by fixing the MOF fragment using DFT with B3LYP-D3 functional and the LACVP**++ basis set.

Paramagnetic NMR Simulations

Spin polarised solid-state hybrid density functional theory (DFT) calculations were performed in the CRYSTAL17 code using the PBE0 hybrid functional.[17,18] A total energy convergence of 2.72×10^{-6} eV was used for all calculations, with a Monkhorst-Pack k-point mesh of 5×5×1 and integral tolerances of 10^{-7}, 10^{-7}, 10^{-7}, 10^{-7}, and 10^{-14}, as defined in the CRYSTAL17 manual.

The structure of $Cu_3(HHTP)_2$ was modelled as a single $Cu_3(HHTP)_2$ layer within the *ab* plane of a periodic unit cell. ~12 Å of vacuum was included along the *c*-axis to separate the layer from its neighbouring image. The atomic positions and unit cell parameters were then optimised to find a low energy configuration. Root-mean-square convergence criteria 8.16×10^{-3} and 3.27×10^{-2} eV were used for maximum gradients and displacements, respectively, during the geometry optimisation. A single Li^+ was then introduced into the $Cu_3(HHTP)_2$ cell adjacent to the Cu node, coordinated by 2 or 3 ACN molecules. A charge neutralising background was applied to the calculations containing Li^+ ions to compensate for the additional positive charge. The atomic positions of all atoms were allowed to optimise under

fixed cell conditions. All-electron POB-triple-zeta valence + polarisation (POB-TZVP-REV2) basis sets were taken from the CRYSTAL online repository and used for Cu, C, O, H, N and Li without further modification.[19]

^{7}Li Fermi contact shifts were calculated from hybrid DFT calculations of the hyperfine coupling constant, A_{iso}, at 0 K using the approach developed in previous studies.[20,21] A_{iso} calculated at 0 K were scaled to finite temperature using a scaling factor, Φ,: [265]

$$\Phi = \frac{B_0 \mu_{\mathrm{eff}}^2}{3k_{\mathrm{B}} g_{\mathrm{e}} \mu_{\mathrm{B}} S_{\mathrm{form}}(T-\theta)} \qquad \text{(S2)}$$

where B_0 is the external magnetic field, k_B is Boltzmann's constant, μ_B is the Bohr magneton and g_e is the free electron g factor. T is experimental temperature, which was assumed to be 320 K due to frictional heating from MAS. S_{form} is the formal spin angular momentum, which is assumed to be S_{form}= 1/2 for Cu^{2+} and $HHTP^{3-}$. μ_{eff} and θ are the effective magnetic moment and Weiss constant, respectively. The value of μ_{eff} and θ were approximated as 1.73 μ_B and 0 K, respectively, equivalent to the Curie spin approximation for a S=1/2 system. These values are in agreement with magnetic measurements of the $Cu_3(HHTP)_2$ system.[22,23]

To isolate the contribution from the unpaired electrons on the Cu^{2+} node, ($\delta^{7\mathrm{Li}}_{\mathrm{node}}$), the spin flipping approach was used in which the Fermi contact shift was initially calculated in the ferromagnetic state ($\delta^{7\mathrm{Li}}_{\mathrm{Ferro}}$) and then the spin on the Cu^{2+} node adjacent to the alkali ion was flipped ($\delta^{7\mathrm{Li}}_{\mathrm{Flipped}}$). The difference in the shift gives the Fermi contact contribution from the node:[21]

$$\delta^{7\mathrm{Li}}_{\mathrm{node}} = \frac{(\delta^{7\mathrm{Li}}_{\mathrm{Ferro}} - \delta^{7\mathrm{Li}}_{\mathrm{Flipped}})}{2}. \qquad \text{(S3)}$$

Figure S1

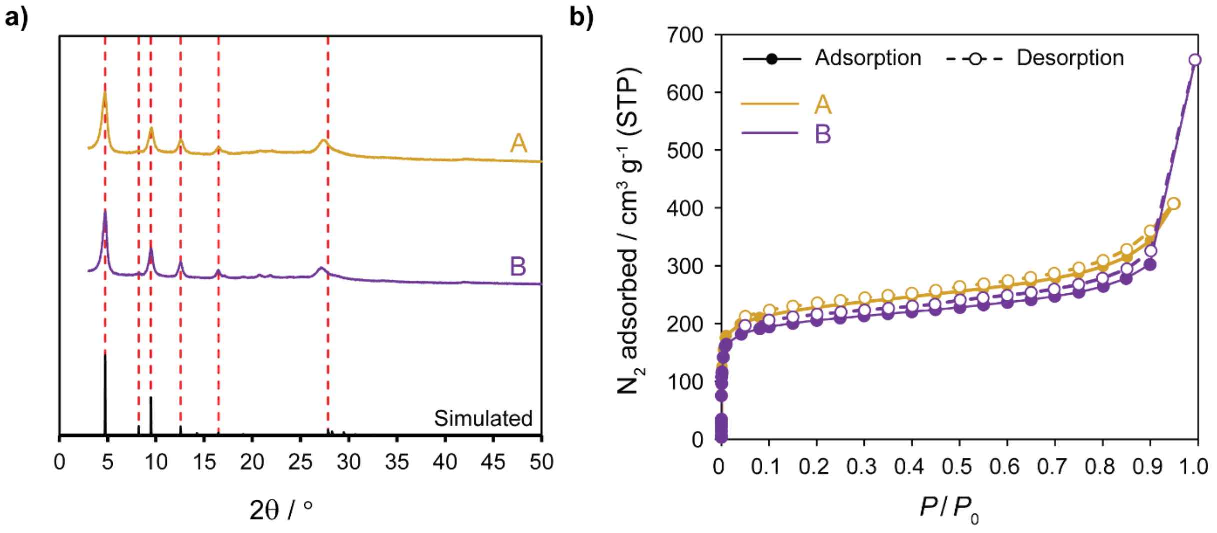

Figure S1: Experimental powder X-Ray diffraction (PXRD) of $Ni_3(HITP)_2$ used in this work, compared to simulated PXRD pattern of a $Ni_3(HITP)_2$ CIF file with eclipsed stacking of the 2D layers along the *c* axis. The comparison confirms identity and crystallinity of the sample used in this work**.** Red dashed lines indicate simulated peak positions based on the theoretical CIF file. **b)** N_2 gas sorption isotherm at 77 K of $Ni_3(HITP)_2$ used in this work.

Figure S2

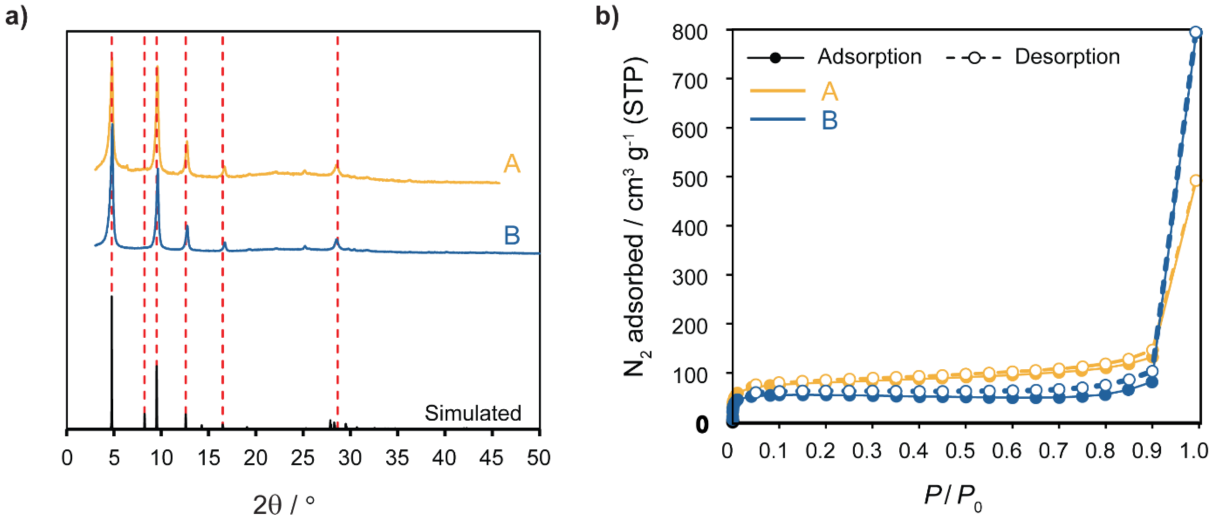

Figure S2: a) Experimental powder X-Ray diffraction (PXRD) of $Zn_3(HHTP)_2$ used in this work, compared to simulated PXRD pattern of a $Ni_3(HITP)_2$ CIF file with eclipsed stacking of the 2D layers along the *c* axis. The comparison confirms identity and crystallinity of the sample used in this work**.** Red dashed lines indicate simulated peak positions based on the theoretical CIF file. A small impurity peak was identified at 25.2 ° and hypothesised to be a form of zinc(II) hydroxide, which was supported by a carbon deficiency in elemental analysis of MOF samples (SI Table S2). **b)** N_2 gas sorption isotherms at 77 K of $Zn_3(HHTP)_2$ used in this work

Figure S3

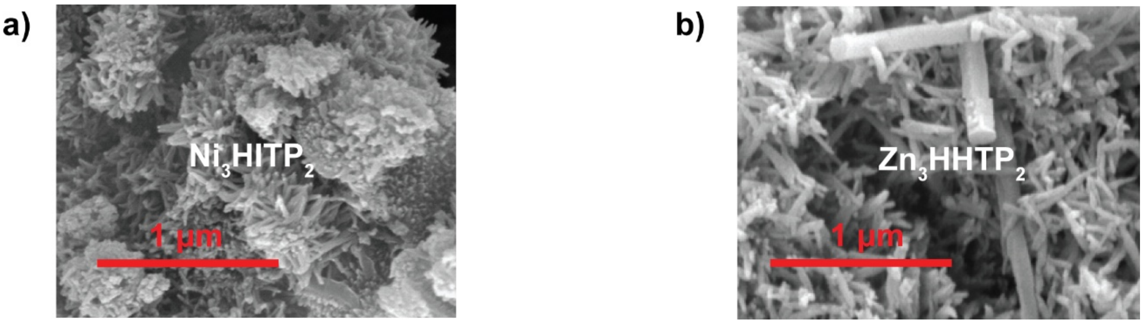

Figure S3: Scanning electron microscopy (SEM) images of selected $Ni_3(HITP)_2$ and $Zn_3(HHTP)_2$ samples. In both cases the particles generally exhibit a rod-like morphology.

Table S2: Predicted & Experimental $Zn_3(HHTP)_2$ Elemental Analysis Results

Element	Predicted / wt. %	Experimental / wt. %
Zn	23.6	23.8
C	51.9	37.6
H	1.5	3.2
N	0.0	0.0

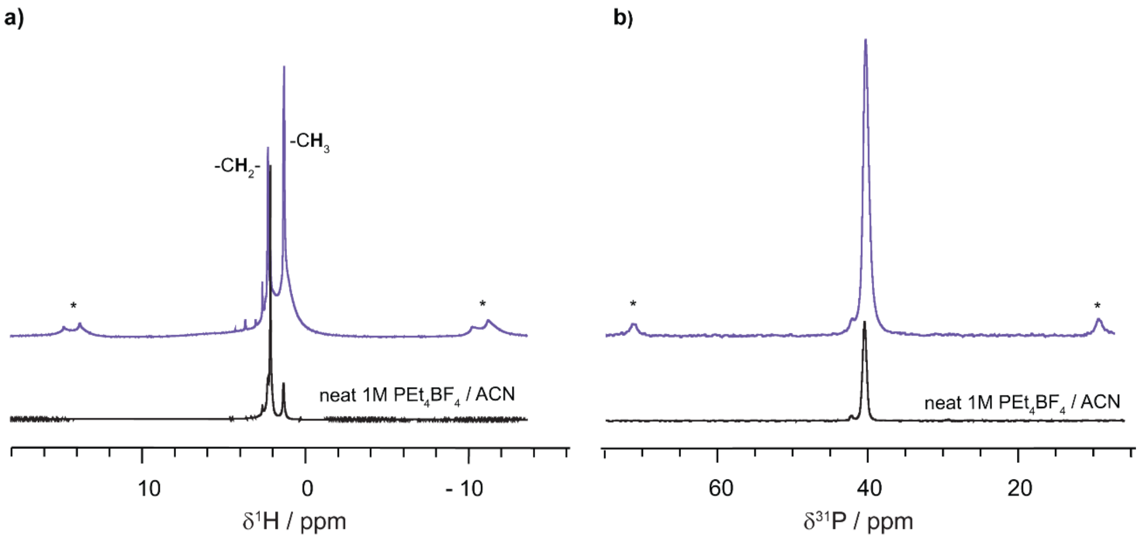

Figure S4: Solid-state NMR spectra at 5 kHz MAS of powder $Zn_3(HHTP)_2$ soaked with 1 M PEt_4BF_4/d_3-ACN **a)** ^1H NMR and **b)** ^{31}P NMR spectrum compared to the neat electrolyte. The ^1H peaks and ^{31}P peak are poorly resolved with minimal shift from the neat electrolyte, making fitting of the cation environments ambiguous. Note that the acetonitrile in the neat electrolyte is un-deuterated, giving rise to a significant acetonitrile peak in the ^1H NMR spectrum.

Figure S5

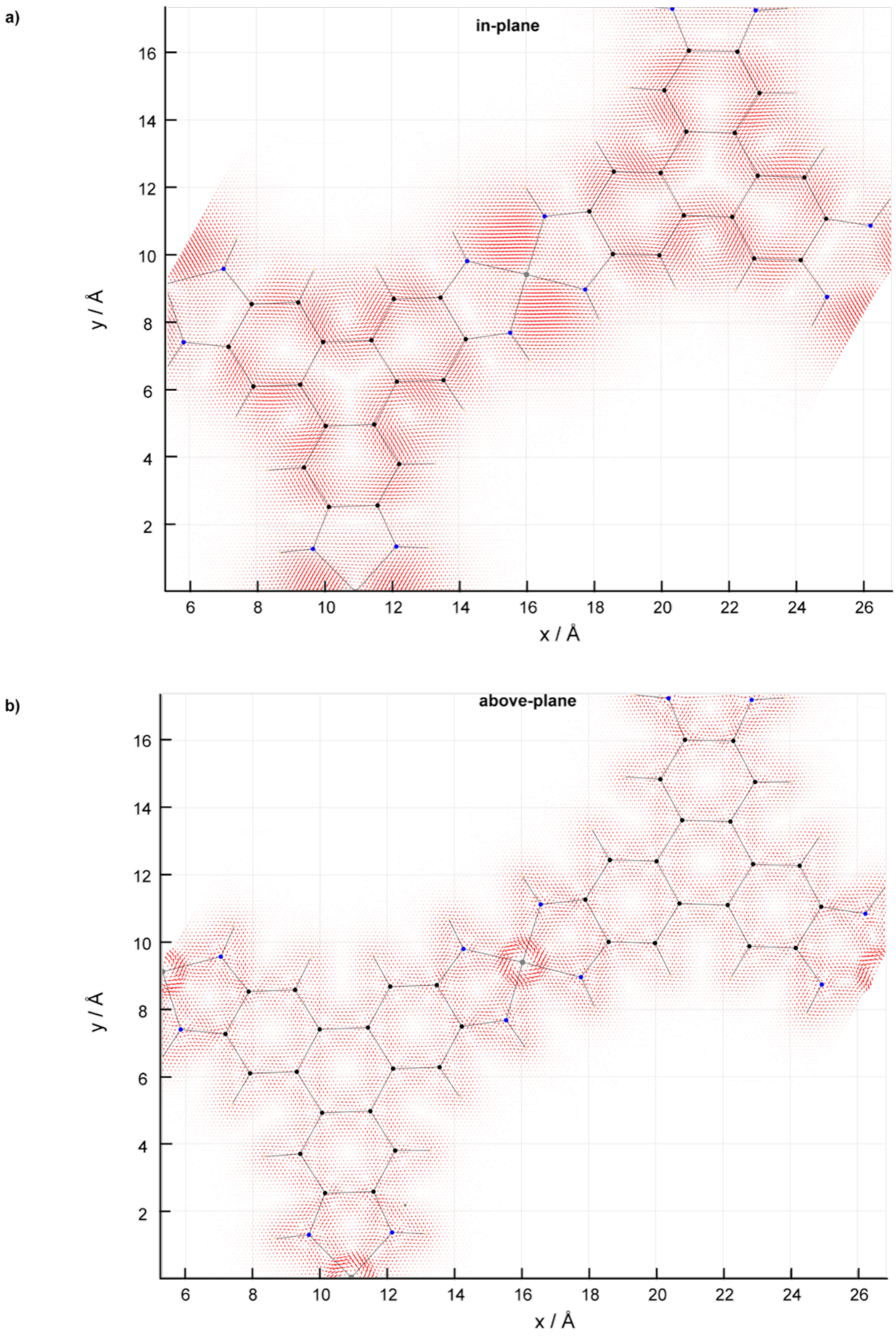

Figure S5: 2D map of induced currents for $Ni_3(HITP)_2$ **a)** in the MOF linker plane and **b)** 2 Å above the plane of the MOF layer, where size of the arrows indicates the magnitude of the current flow. Larger arrows indicate strong current flow around metal node. There is a significant ring-current from the aromatic linkers, as well as an induced current contribution from the metal.

Figure S6

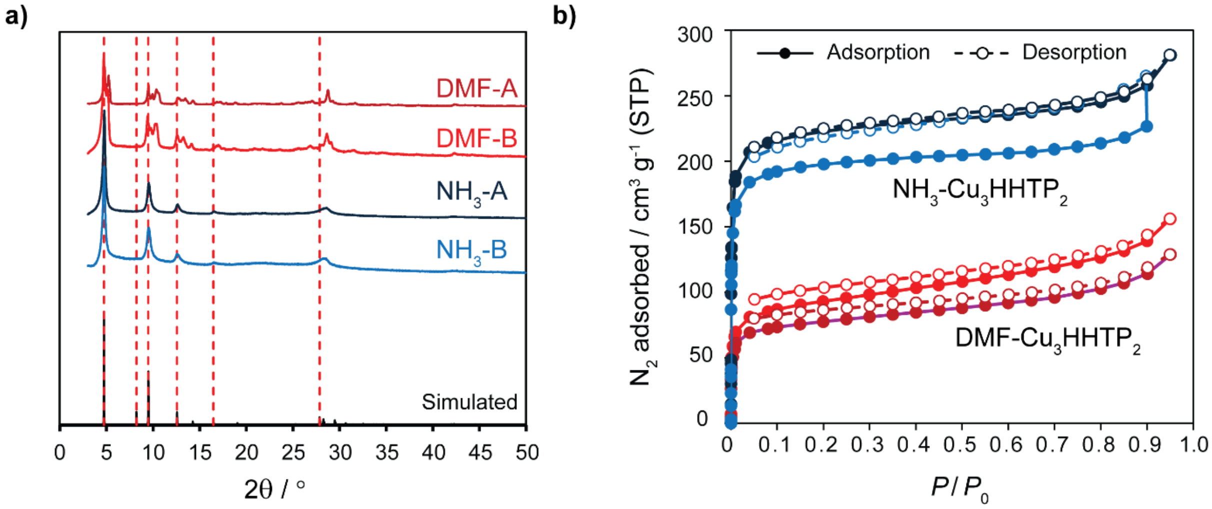

Figure S6: Red represents DMF-modulated $Cu_3(HHTP)_2$ samples which have a rod-like morphology; blue represents NH_3-modulated $Cu_3(HHTP)_2$ samples, which have a flake-like morphology. **a)** Experimental powder X-Ray diffraction (PXRD) of $Cu_3(HHTP)_2$ used in this work, compared to simulated PXRD pattern of a $Ni_3(HITP)_2$ CIF file with eclipsed stacking of the 2D layers along the *c* axis, where the red dashed lines indicate the theoretical peak positions. Note that the layers are not totally eclipsed in the MOFs[3], so different degrees of disorder in the stacking of the layers leads to a different diffraction pattern for the two morphologies. Our diffraction patterns are similar to those in previously published work for these two materials.[24,25] **b)** N_2 gas sorption isotherm at 77 K of $Cu_3(HHTP)_2$ used in this work. The rod-like morphology is more susceptible to pore blockages, which leads to a lower microporosity in the N_2 sorption isotherm.

Table S3: Diamagnetic ^{7}Li NMR Chemical Shift Calculations

**Electrolyte**	**MOF**	***δ*** **^{7}Li (in-pore) / ppm**	***δ*** **^{7}Li (electrolyte) / ppm**	**Δ*δ*** **^{19}F / ppm**
LiTFSI/ACN	$Zn_3(HHTP)_2$	−2.7	−3.3	+0.6
	$Cu_3(HHTP)_2$	−3.8	−3.3	−0.5

Figure S7

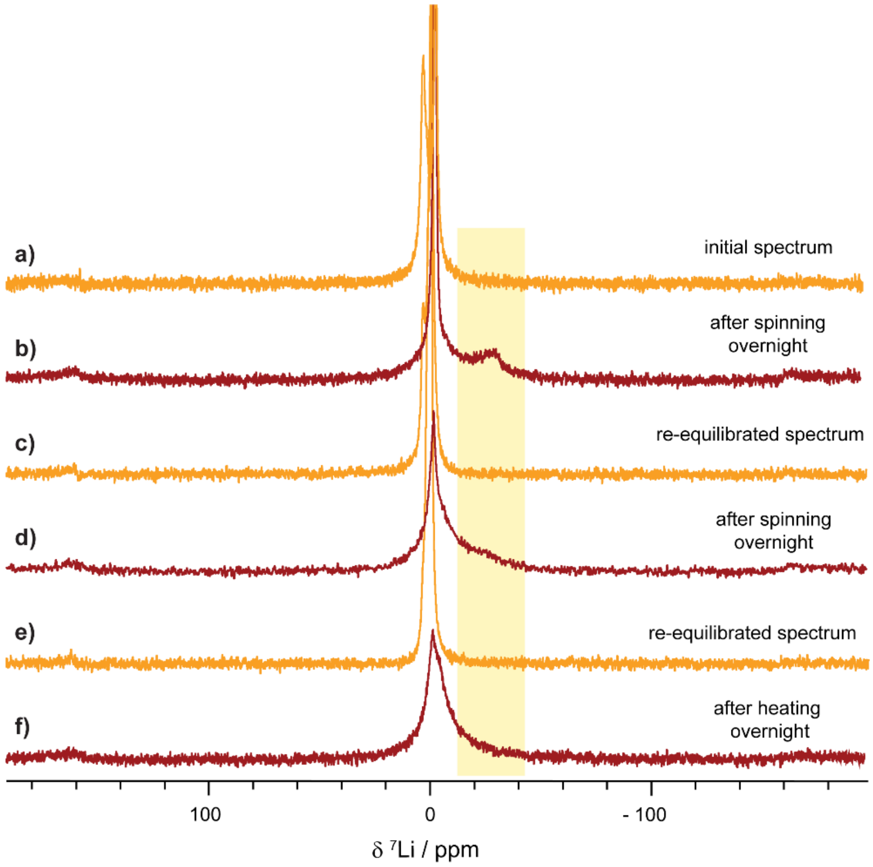

Figure S7: ^{7}Li solid-state NMR (9.4 T) experiments at 25 kHz MAS of DMF-$Cu_3(HHTP)_2$ Sample A, soaked with 1 M LiTFSI in ACN electrolyte. Spectra are quantitative. Spectra a–f represent the chronological order of the spectra recorded. **a)** Initial spectrum **b)** Spectrum after spinning sample overnight **c)** Spectrum after the sample was left for 2 weeks to re-equilibrate **d)** Spectrum after spinning the sample again overnight **e)** Spectrum after the sample was left for 1 week to re-equilibrate **f)** Spectrum after heating the rotor in an oven overnight. The yellow highlighted region shows the appearance of a negatively shifted paramagnetic peak from heating (through fast spinning for a prolonged period).

Figure S8

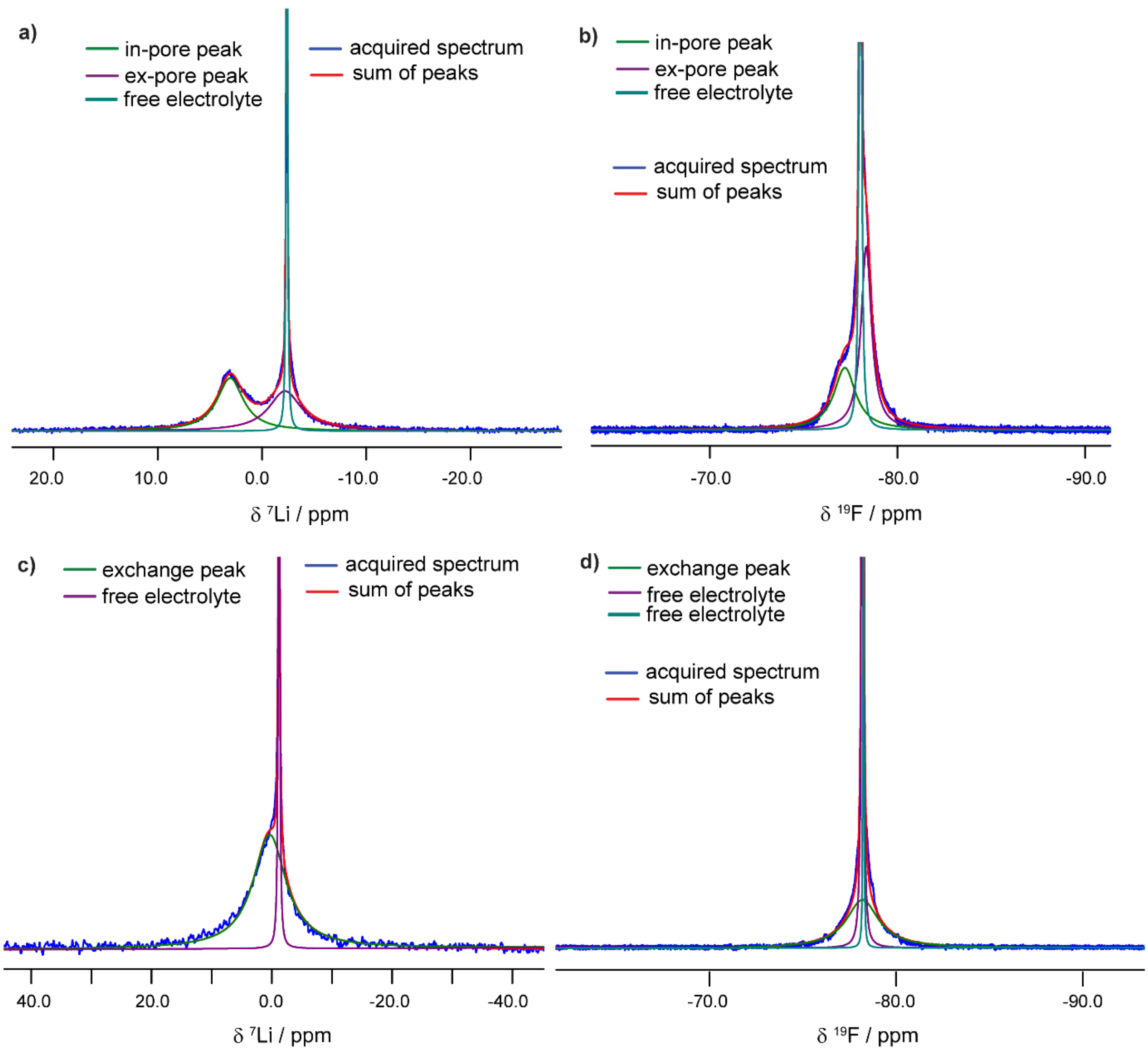

Figure S8: Example deconvolution of ^{7}Li and ^{19}F solid-state NMR spectra at 25 kHz MAS of **a), b)** DMF-$Cu_3(HHTP)_2$ rods Sample A and **c), d)** NH_3-$Cu_3(HHTP)_2$ flakes Sample A, soaked with 1 M LiTFSI in acetonitrile electrolyte, spectra from Figure 6.

Figure S9

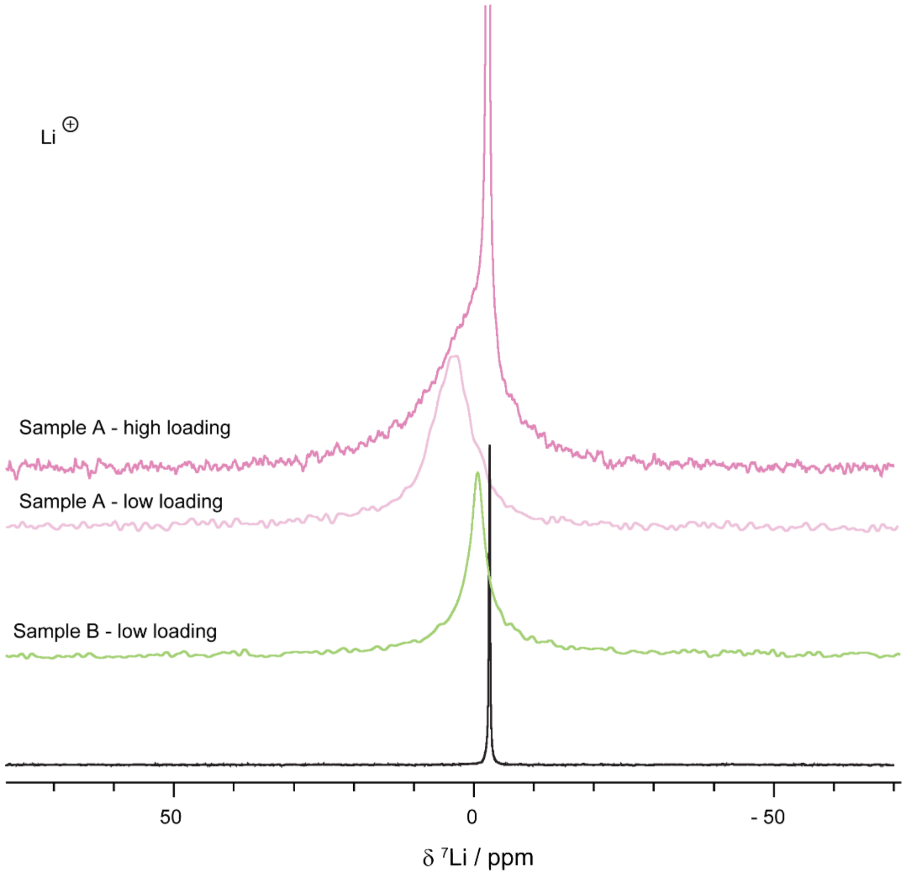

Figure S9: Solid-state quantitative ^{7}Li NMR (9.4 T) experiments at 25 kHz MAS of NH_3-$Cu_3(HHTP)_2$ flakes samples soaked with a various loadings of 1 M LiTFSI/ACN electrolyte, compared with NMR spectrum of neat electrolyte. Depending on loading and sample, various line-shapes are observed.

Figure S10

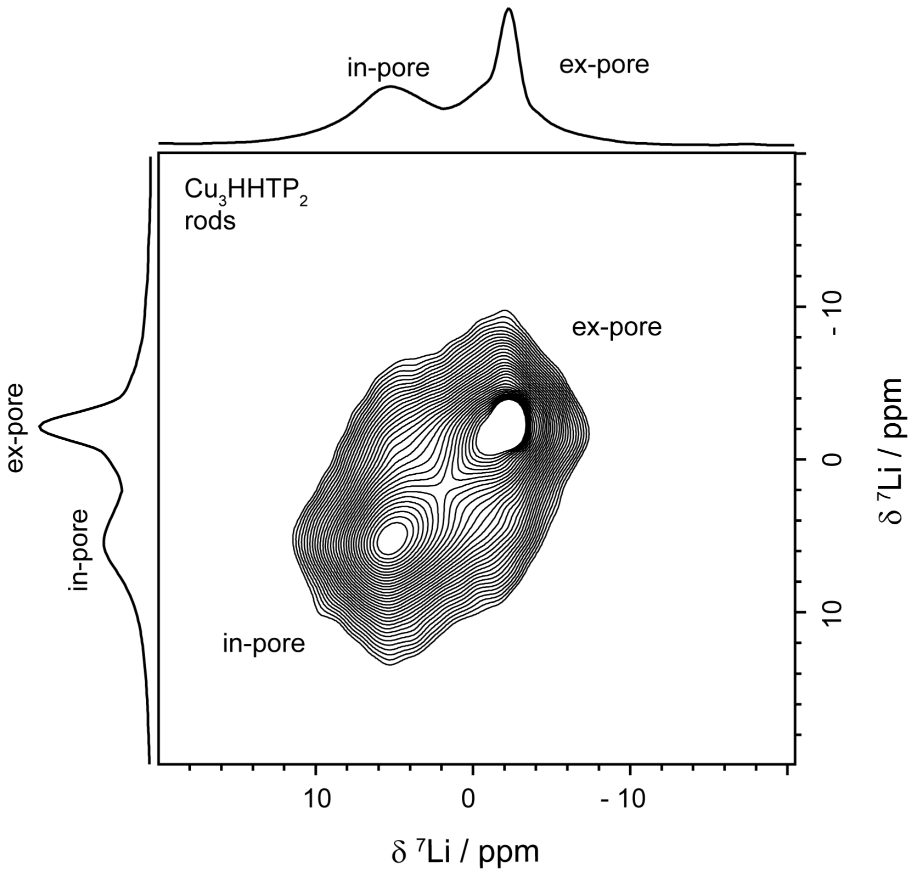

Figure S10: ^{7}Li solid-state EXSY NMR (9.4 T) experiments at 25 kHz MAS of DMF-$Cu_3(HHTP)_2$ Sample B with 1 M LiTFSI in ACN electrolyte, with a mixing time of 0.5 ms. An absence of cross peaks is shown at this low mixing time compared to Figure 7a.

Figure S11

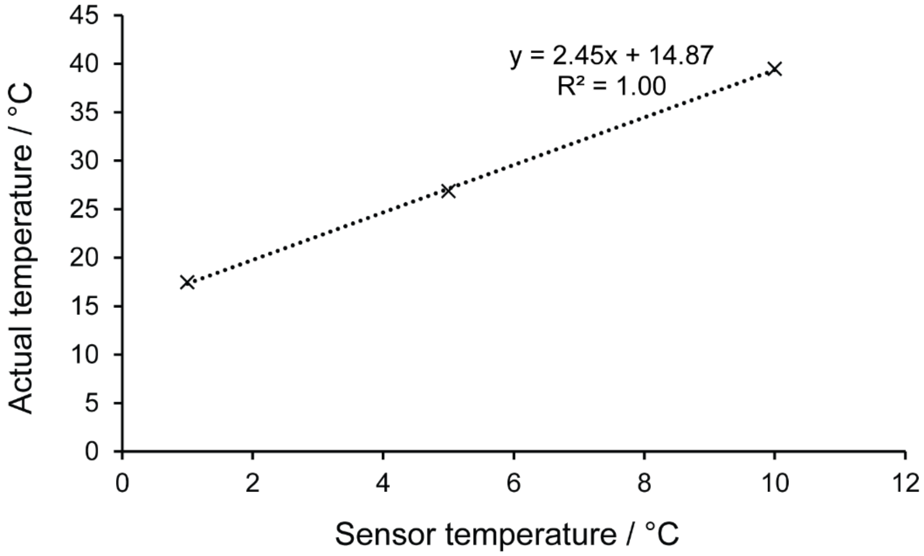

Figure S11: Temperature calibration curve at a MAS rate of 25 kHz from an inversion-recovery ^{79}Br pulse sequence applied to KBr, used to calibrate the temperature in Figure 5.

Discussion on Exchange

The exchange differences observed for the two $Cu_3(HHTP)_2$ morphologies in Figures 6 and 7 can be explained as a result of the approximately 100 times smaller length-to-width aspect ratio of the flake-like morphology compared to the rod-like morphology.[3] This allows electrolyte to move more freely between in-pore and ex-pore environments due to short pore channels with many pore openings accessible for $Cu_3(HHTP)_2$ flakes, compared to long pore channels with few pore openings for $Cu_3(HHTP)_2$ rods.[3] The critical exchange time can be approximated by the inverse of the in-pore/ex-pore peak separation in Hz. Assuming a similar self-diffusion coefficient of in-pore ions as measured for porous carbon, $D_{\text{in-pore}}$~10^{-12} m^2 s^{-1}, the root-mean square displacement (RMSD) of atomic positions corresponding to diffusion of electrolyte in the MOF pores can be estimated.[26] Considering 1D diffusion along the pore channels, this can be estimated by $\sqrt{2D_{\text{in-pore}}t}$, where t is characteristic timescale for exchange.[26] Environments significantly closer than this critical distance will exchange on the NMR timescale and can be expected to be in fast-exchange, whereas environments for which the separation significantly exceeds this distance are expected to be in slow-exchange, with intermediate exchange between. Depending on the measured $\Delta\delta$ for $Cu_3(HHTP)_2$ rods, which varies between spectra, the corresponding limiting RMSD is on the order of 40-70 nm. Whilst the length of $Cu_3(HHTP)_2$ rods (0.5 – 4 µm) generally exceeds this, the thickness of the $Cu_3(HHTP)_2$ flakes (30 – 110 nm) is a borderline scenario, spanning across the range.[3] Meaning, whilst on the NMR timescale electrolyte ions may move in and out of the pores along the short axis of the $Cu_3(HHTP)_2$ flakes, they may not exit the pores running along the $Cu_3(HHTP)_2$ rods. As such, in-pore and ex-pore environments in $Cu_3(HHTP)_2$ rods are expected to be viewed in a slow exchange regime, whilst intermediate-fast exchange is expected between environments in $Cu_3(HHTP)_2$ flakes. If the broad peak in $Cu_3(HHTP)_2$ flakes is considered as the in-pore peak rather than an intermediate exchange environment, the measured $\Delta\delta$ is significantly lower relative to those measured for $Cu_3(HHTP)_2$ rods for both cations and anions (**Figure 6c**). Given the two MOFs would be expected to have the same $\Delta\delta$, this supports the hypothesis that this broad peak observed for $Cu_3(HHTP)_2$ flakes instead represents a weighted average from exchange of the in-pore and ex-pore environments, therefore reducing the observed $\Delta\delta$.

References

(1) Balhatchet, C. J.; Gittins, J. W.; Shin, S.-J.; Ge, K.; Liu, X.; Trisukhon, T.; Sharma, S.; Kress, T.; Taberna, P.-L.; Simon, P.; Walsh, A.; Forse, A. C. Revealing Ion Adsorption and Charging Mechanisms in Layered Metal–Organic Framework Supercapacitors with Solid-State Nuclear Magnetic Resonance. *J. Am. Chem. Soc.* **2024**, *146* (33), 23171–23181. https://doi.org/10.1021/jacs.4c05330.

(2) Misumi, Y.; Yamaguchi, A.; Zhang, Z.; Matsushita, T.; Wada, N.; Tsuchiizu, M.; Awaga, K. Quantum Spin Liquid State in a Two-Dimensional Semiconductive Metal–Organic Framework. *J. Am. Chem. Soc.* **2020**, *142* (39), 16513–16517. https://doi.org/10.1021/jacs.0c05472.

(3) Gittins, J. W.; Balhatchet, C. J.; Fairclough, S. M.; Forse, A. C. Enhancing the Energy Storage Performances of Metal–Organic Frameworks by Controlling Microstructure. *Chem. Sci.* **2022**, *13* (32), 9210–9219. https://doi.org/10.1039/D2SC03389E.

(4) Gittins, J. W.; Ge, K.; Balhatchet, C. J.; Taberna, P.-L.; Simon, P.; Forse, A. C. Understanding Electrolyte Ion Size Effects on the Performance of Conducting Metal–Organic Framework Supercapacitors. *J. Am. Chem. Soc.* **2024**, *146* (18), 12473–12484. https://doi.org/10.1021/jacs.4c00508.

(5) Momma, K.; Izumi, F. *VESTA 3*for Three-Dimensional Visualization of Crystal, Volumetric and Morphology Data. *J. Appl. Crystallogr.* **2011**, *44* (6), 1272–1276. https://doi.org/10.1107/s0021889811038970.

(6) Thommes, M.; Kaneko, K.; Neimark, A. V.; Olivier, J. P.; Rodriguez-Reinoso, F.; Rouquerol, J.; Sing, K. S. W. Physisorption of Gases, with Special Reference to the Evaluation of Surface Area and Pore Size Distribution (IUPAC Technical Report). *Pure Appl. Chem.* **2015**, *87* (9–10), 1051–1069. https://doi.org/10.1515/pac-2014-1117.

(7) Beckett, P.; Denning, M. S.; Carravetta, M.; Kalda, A.; Heinmaa, I. Field Dependence of the Relaxation of 79Br in KBr and Its Use as a Temperature Calibrant. *J. Magn. Reson.* **2012**, *223*, 61–63. https://doi.org/10.1016/j.jmr.2012.07.007.

(8) Massiot, D.; Fayon, F.; Capron, M.; King, I.; Le Calvé, S.; Alonso, B.; Durand, J.; Bujoli, B.; Gan, Z.; Hoatson, G. Modelling One- and Two-dimensional Solid-state NMR Spectra. *Magn. Reson. Chem.* **2002**, *40* (1), 70–76. https://doi.org/10.1002/mrc.984.

(9) Perdew, J. P.; Burke, K.; Ernzerhof, M. Generalized Gradient Approximation Made Simple. *Phys. Rev. Lett.* **1996**, *77* (18), 3865–3868. https://doi.org/10.1103/PhysRevLett.77.3865.

(10) Perdew, J. P.; Ernzerhof, M.; Burke, K. Rationale for Mixing Exact Exchange with Density Functional Approximations. *J. Chem. Phys.* **1996**, *105* (22), 9982–9985. https://doi.org/10.1063/1.472933.

(11) Grimme, S.; Antony, J.; Ehrlich, S.; Krieg, H. A Consistent and Accurate *Ab Initio* Parametrization of Density Functional Dispersion Correction (DFT-D) for the 94 Elements H-Pu. *J. Chem. Phys.* **2010**, *132* (15), 154104. https://doi.org/10.1063/1.3382344.

(12) Pickard, C. J.; Mauri, F. All-Electron Magnetic Response with Pseudopotentials: NMR Chemical Shifts. *Phys. Rev. B - Condens. Matter Mater. Phys.* **2001**, *63* (24), 2451011–2451013. https://doi.org/10.1103/physrevb.63.245101.

(13) Yates, J. R.; Pickard, C. J.; Mauri, F. Calculation of NMR Chemical Shifts for Extended Systems Using Ultrasoft Pseudopotentials. *Phys. Rev. B - Condens. Matter Mater. Phys.* **2007**, *76* (2), 1–11. https://doi.org/10.1103/PhysRevB.76.024401.

(14) Shin, S.-J.; Gittins, J. W.; Golomb, M. J.; Forse, A. C.; Walsh, A. Microscopic Origin of Electrochemical Capacitance in Metal–Organic Frameworks. *J. Am. Chem. Soc.* **2023**, *145* (26), 14529–14538. https://doi.org/10.1021/jacs.3c04625.

(15) Valiev, M.; Bylaska, E. J.; Govind, N.; Kowalski, K.; Straatsma, T. P.; Van Dam, H. J. J.; Wang, D.; Nieplocha, J.; Apra, E.; Windus, T. L.; de Jong, W. A. NWChem: A Comprehensive and Scalable Open-Source Solution for Large Scale Molecular Simulations. *Comput. Phys. Commun.* **2010**, *181* (9), 1477–1489. https://doi.org/10.1016/j.cpc.2010.04.018.

(16) Dupuis, M. New Integral Transforms for Molecular Properties and Application to a Massively Parallel GIAO-SCF Implementation. *Comput. Phys. Commun.* **2001**, *134* (2), 150–166. https://doi.org/10.1016/S0010-4655(00)00195-8.

(17) Dovesi, R.; Erba, A.; Orlando, R.; Zicovich-Wilson, C. M.; Civalleri, B.; Maschio, L.; Rérat, M.; Casassa, S.; Baima, J.; Salustro, S.; Kirtman, B. Quantum-mechanical Condensed Matter Simulations with CRYSTAL. *WIREs Comput. Mol. Sci.* **2018**, *8* (4), e1360. https://doi.org/10.1002/wcms.1360.

(18) Adamo, C.; Barone, V. Toward Reliable Density Functional Methods without Adjustable Parameters: The PBE0 Model. *J. Chem. Phys.* **1999**, *110* (13), 6158–6170. https://doi.org/10.1063/1.478522.

(19) Vilela Oliveira, D.; Laun, J.; Peintinger, M. F.; Bredow, T. BSSE-correction Scheme for Consistent Gaussian Basis Sets of Double- and Triple-zeta Valence with Polarization Quality for Solid-state Calculations. *J. Comput. Chem.* **2019**, *40* (27), 2364–2376. https://doi.org/10.1002/jcc.26013.
(20) Kim, J.; Middlemiss, D. S.; Chernova, N. A.; Zhu, B. Y. X.; Masquelier, C.; Grey, C. P. Linking Local Environments and Hyperfine Shifts: A Combined Experimental and Theoretical^{31} P and^{7} Li Solid-State NMR Study of Paramagnetic Fe(III) Phosphates. *J. Am. Chem. Soc.* **2010**, *132* (47), 16825–16840. https://doi.org/10.1021/ja102678r.
(21) Middlemiss, D. S.; Ilott, A. J.; Clément, R. J.; Strobridge, F. C.; Grey, C. P. Density Functional Theory-Based Bond Pathway Decompositions of Hyperfine Shifts: Equipping Solid-State NMR to Characterize Atomic Environments in Paramagnetic Materials. *Chem. Mater.* **2013**, *25* (9), 1723–1734. https://doi.org/10.1021/cm400201t.
(22) Ninawe, P.; Jain, A.; Sangole, M.; Anas, M.; Ugale, A.; Malik, V. K.; Yusuf, S. M.; Singh, K.; Ballav, N. Robust Spin Liquidity in 2D Metal-Organic Framework Cu_3 $(HHTP)_2$ with S=$^1/_2$ Kagome Lattice. *Chem. – Eur. J.* **2024**, *30* (4), e202303718. https://doi.org/10.1002/chem.202303718.
(23) Misumi, Y.; Yamaguchi, A.; Zhang, Z.; Matsushita, T.; Wada, N.; Tsuchiizu, M.; Awaga, K. Quantum Spin Liquid State in a Two-Dimensional Semiconductive Metal–Organic Framework. *J. Am. Chem. Soc.* **2020**, *142* (39), 16513–16517. https://doi.org/10.1021/jacs.0c05472.
(24) Hoppe, B.; Hindricks, K. D. J.; Warwas, D. P.; Schulze, H. A.; Mohmeyer, A.; Pinkvos, T. J.; Zailskas, S.; Krey, M. R.; Belke, C.; König, S.; Fröba, M.; Haug, R. J.; Behrens, P. Graphene-like Metal–Organic Frameworks: Morphology Control, Optimization of Thin Film Electrical Conductivity and Fast Sensing Applications. *CrystEngComm* **2018**, *20* (41), 6458–6471. https://doi.org/10.1039/C8CE01264D.
(25) Day, R. W.; Bediako, D. K.; Rezaee, M.; Parent, L. R.; Skorupskii, G.; Arguilla, M. Q.; Hendon, C. H.; Stassen, I.; Gianneschi, N. C.; Kim, P.; Dincă, M. Single Crystals of Electrically Conductive Two-Dimensional Metal–Organic Frameworks: Structural and Electrical Transport Properties. *ACS Cent. Sci.* **2019**, *5* (12), 1959–1964. https://doi.org/10.1021/acscentsci.9b01006.
(26) Forse, A. C.; Griffin, J. M.; Merlet, C.; Carretero-Gonzalez, J.; Raji, A.-R. O.; Trease, N. M.; Grey, C. P. Direct Observation of Ion Dynamics in Supercapacitor Electrodes Using in Situ Diffusion NMR Spectroscopy. *Nat. Energy* **2017**, *2* (3), 16216. https://doi.org/10.1038/nenergy.2016.216.
